# Buffering Volume Change in Solid-State Battery Composite Cathodes with CO_2_-Derived Block Polycarbonate Ethers

**DOI:** 10.1021/jacs.2c06138

**Published:** 2022-09-19

**Authors:** Georgina L. Gregory, Hui Gao, Boyang Liu, Xiangwen Gao, Gregory J. Rees, Mauro Pasta, Peter G. Bruce, Charlotte K. Williams

**Affiliations:** † Chemistry Research Laboratory, University of Oxford, 12 Mansfield Road, Oxford OX1 3TA, U.K.; ‡ Department of Materials, University of Oxford, Parks Road, Oxford OX1 3PH, U.K.

## Abstract

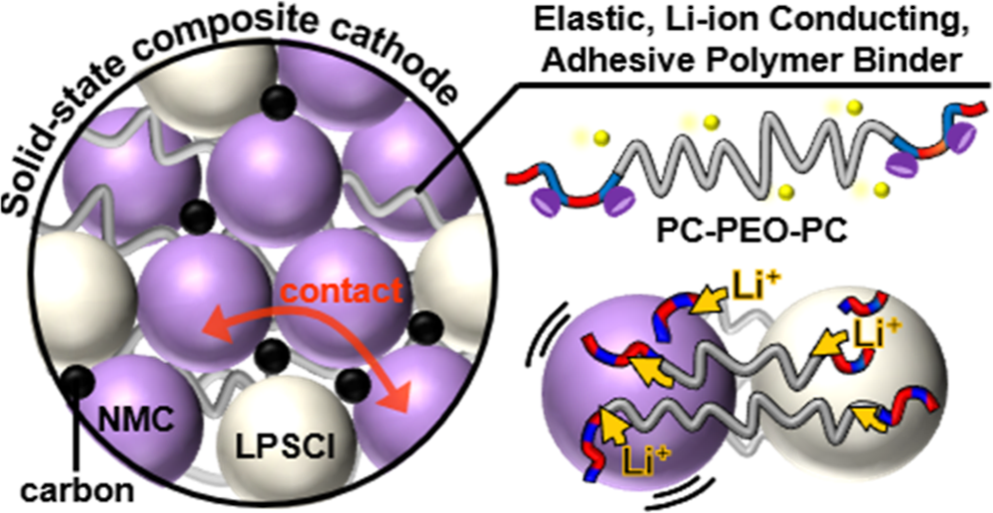

Polymers designed with a specific combination of electrochemical, mechanical, and chemical properties could help overcome challenges limiting practical all-solid-state batteries for high-performance next-generation energy storage devices. In composite cathodes, comprising active cathode material, inorganic solid electrolyte, and carbon, battery longevity is limited by active particle volume changes occurring on charge/discharge. To overcome this, impractical high pressures are applied to maintain interfacial contact. Herein, block polymers designed to address these issues combine ionic conductivity, electrochemical stability, and suitable elastomeric mechanical properties, including adhesion. The block polymers have “hard-soft-hard”, ABA, block structures, where the soft “B” block is poly(ethylene oxide) (PEO), known to promote ionic conductivity, and the hard “A” block is a CO_2_-derived polycarbonate, poly(4-vinyl cyclohexene oxide carbonate), which provides mechanical rigidity and enhances oxidative stability. ABA block polymers featuring controllable PEO and polycarbonate lengths are straightforwardly prepared using hydroxyl telechelic PEO as a macroinitiator for CO_2_/epoxide ring-opening copolymerization and a well-controlled Mg(II)Co(II) catalyst. The influence of block polymer composition upon electrochemical and mechanical properties is investigated, with phosphonic acid functionalities being installed in the polycarbonate domains for adhesive properties. Three lead polymer materials are identified; these materials show an ambient ionic conductivity of 10 ^–4^ S cm^–1^, lithium-ion transport (*t*_Li+_ 0.3–0.62), oxidative stability (>4 V vs Li^+/^Li), and elastomeric or plastomer properties (*G*′ 0.1–67 MPa). The best block polymers are used in composite cathodes with LiNi_0.8_Mn_0.1_Co_0.1_O_2_ active material and Li_6_PS_5_Cl solid electrolyte–the resulting solid-state batteries demonstrate greater capacity retention than equivalent cells featuring no polymer or commercial polyelectrolytes.

## Introduction

All-solid-state batteries (SSBs) offer one of the few routes for the implementation of lithium anodes and therefore a step change in energy density compared with current rechargeable lithium-ion batteries based on liquid electrolytes.^1^ Using non-flammable solid-state electrolytes can also deliver improvements in safety, important to address the stringent requirements for deployment in electric vehicles and large-scale energy storage.^2^ Solid-state sulfide-based electrolytes, such as Li_6_PS_5_Cl (LPSCl; argyrodite), exhibit high ionic conductivities (2–5 mS cm^–1^ at room temperature, RT) and show suitable mechanical properties and processability for large-scale device fabrication.^3^ The composite cathodes must comprise an intimate mixture of active inorganic cathode material, solid electrolyte, and carbon–the challenge is to mimic the cathode surface wetting achieved by liquid electrolytes.^4^ Even if excellent physical mixing can be achieved, the cathode volume changes, and the resulting cell mechanical forces tend to limit battery cycling.^5^ For example, a leading high-voltage cathode material, LiNi_0.8_Mn_0.1_Co_0.1_O_2_ (NMC811), experiences ∼6% volume change during every cycle of battery charge/discharge as lithium is inserted/extracted.^6^ Over many cycles, this volumetric strain reduces particle–particle interfacial contacts and accelerates battery failure.^7^ Although the delamination may be prevented by holding the cell under very high pressures (∼50 MPa), such solutions are impractical for many applications.^8^

Elastomeric polymers could compensate for the inorganic active-phase volume changes.^9^ Commercial elastomers like styrenic block copolymers (SBCs) or nitrile butadiene rubbers (NBRs) were used in composite cathodes, showing improved capacity retention in the resulting SSBs.^10^ Nonetheless, these low-polarity hydrocarbon polymers have poor attachment to the inorganic electrodes and form rather unstable interfaces, which may undergo premature contact failure. Computational work, by Carter and co-workers, suggests that interfacial delamination is induced when electrode particles undergo as little as 7.5% volume change during (de)lithiation.^11^ Polymer elastomers featuring functional substituents, for example, capable of hydrogen bonding, may show better compatibility with the inorganic materials.^10a,12^ SBC modified with 10% carboxylic acid groups showed 1.4 × greater adhesion to NbO-coated NMC compared with NBR binders, resulting in cells showing 20% greater capacity retention.^13^ Generally, the polymer elastomers are non-conductive, so they cannot facilitate ionic transport in the composite cathode.^14^ An attractive solution would be to design polymers for SSBs that combine ionic conductivity, electrochemical stability, interfacial adhesion, and suitable mechanical properties.^15^ Recent work has demonstrated improvements in composite cathode performance using a polytetrafluroethylene (PTFE)-based binder modified to impart moderate lithium-ion conductivity (1.5 × 10^–5^ S cm^–1^ at RT).^16^

Here, our strategy is to target well-defined ABA-type triblock polymers comprising polycarbonate (PC) and poly(ethylene oxide) (PEO) blocks. PEO was chosen as the “B” mid-segment as it is perhaps the most successful ionically conductive polymer to date due to its unrivalled ability to solvate various lithium salts.^17^ Ion transport, by complexation to Li ions and hopping between oxygen atoms, is facilitated by its high chain flexibility and related to its low glass transition temperature (*T*_g_ ∼ −64 °C).^18^ Block copolymers are well known to phase-separate into predictable nanostructures, and this can be exploited to tune mechanical properties.^19^ A good example is polymer electrolytes based on phase-separated poly(styrene (PS)-*b*-PEO), with lithium bis(trifluoromethanesulfonyl)imide (LiTFSI). The PS block delivers a high Young’s modulus (∼3 GPa) and *T*_g_ (∼90 °C), thereby imparting mechanical stability, while the PEO phase retains lithium-ion conductivity (∼10^–5^ S cm^–1^ at RT).^20^ Although a very promising strategy for some cells, we posit that such materials would be less effective in composite cathodes since PS is non-polar and not conductive; hence, its inorganic surface adhesion would be low. Here, we designed block polymers to feature outer A blocks, which are rigid polycarbonates (*T*_g_ ∼ 100 °C) prepared by the alternating ring-opening copolymerization (ROCOP) of CO_2_ with 4-vinyl cyclohexene oxide (vCHO). In addition to providing network sites for optimizing mechanical properties, these blocks should maximize conductivity and inorganic material compatibility since they are oxygenated and polar. The direct incorporation of CO_2_ as a raw material may also be desirable from a sustainable raw material viewpoint. The use of well-controlled ROCOP chemistry allows for excellent control over composition, molar mass, and chain end groups to moderate ion transport properties.^21^ The vinyl substituent, present on each A-block repeat unit, also allows for the installation of chemical functionalities to tailor interfacial adhesion. Here, phosphonic acid groups are installed as ligands for the inorganic oxide surfaces; the groups are attached to the polymer backbone using high-efficiency thiol–ene reactions.

Some polycarbonate electrolytes have been noted to show superior oxidative stabilities (4.5–5 V) than polyethers (<3.5 V).^22^ Such stability is important in composites employing high-voltage cathodes. The lower ionic conductivities of polycarbonate electrolytes, compared to PEO, prompted investigation of poly(ether-carbonates) showing improved electrochemical properties.^23^ These studies applied less well-defined polymers or random copolymers, and generally, the polymer mechanical properties were not reported or the materials required permanent chemical cross-linking to achieve mechanical integrity–both strategies limit processability.^24^ In the present investigation, poly(carbonate-*b*-ether-*b*-carbonates) are developed to deliver ionically conductive, adhesive elastomers, specifically designed to compensate for cathode volume changes using NMC and LPSCl since these inorganics are among the best performing cathodes and solid-state electrolytes, respectively.

## Results and Discussion

### Polymer Synthesis

**Figure 1:**
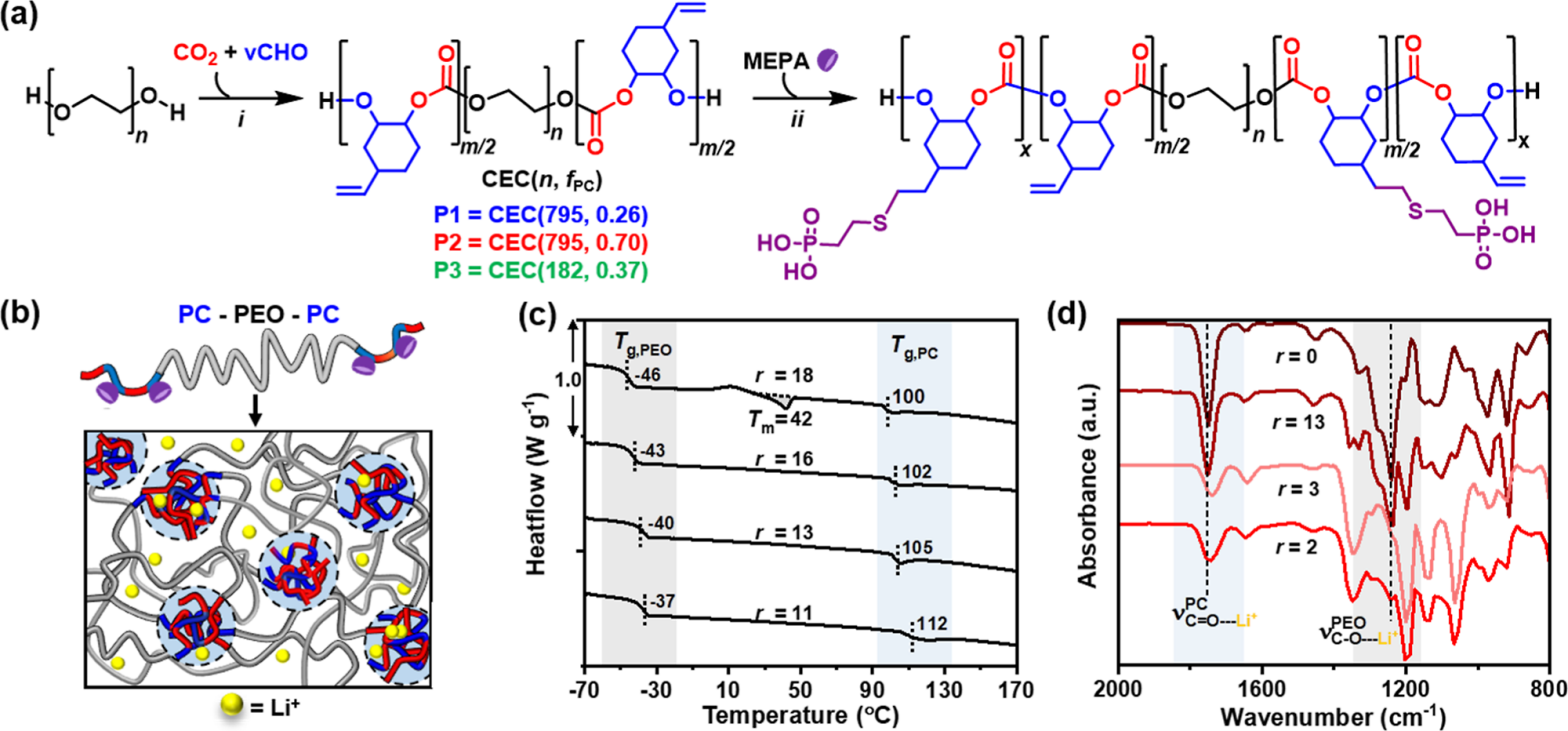
(a) Reaction scheme: (i) CO_2_/vCHO ROCOP using PEO macroinitiator (Table S2). (ii) UV-mediated thiol–ene reaction with 2-mercaptoethyl phosphonic acid (MEPA). (b) Schematic of phase-separated PC/PEO blocks with lithium salt (anions not shown). (c) DSC data for **P1** with different LiTFSI ratios {*r* = [EO + CO]/[Li]}. (d) FTIR spectra for **P2** with different salt ratios.

Triblock polymers were synthesized using commercial hydroxyl-telechelic PEO samples featuring 23-2272 EO repeat units. This corresponds to molar masses of 1–100 kg mol^–1^ and dispersities of 1.08–1.13 (Table S1). These bifunctional macroinitiators were applied in the ring-opening copolymerization of CO_2_ with vCHO, catalyzed by a high-activity heterodinuclear complex [LMgCo(OAc)_2_] (Figure 1a, see Supporting Information for the catalyst structure and ROCOP mechanism).^25^ This catalyst has previously shown high activity for CHO/CO_2_ ROCOP (TOF = 455–1205 h^–1^ at 80–120 °C) and is very selective for carbonate linkages. Polymerizations were conducted at 1 bar CO_2_ pressure, on a 10–15 g scale, in neat epoxide or diluted with diethyl carbonate to moderate viscosity. Successful conversion to the ABA block structure was determined by ^1^H NMR spectroscopy of the crude polymers. PC-*b*-PEO-*b*-PC samples were isolated (by precipitation from diethyl ether) as white powders in high yield (85–90%). Repeated precipitations were conducted to remove any unreacted monomer or catalyst residue. Any trace metals remaining (<10 ppm) are unlikely to influence battery performance. The block polymer structure was verified by multiple characterization methods (Figures S2–S4).^26^ Size-exclusion chromatography (SEC) confirmed an increase in polymer molecular mass (*M*_n_), compared with that of the PEO macroinitiator, and the block polymers maintained narrow dispersity (*Đ* ∼ 1.13–1.23). Polymer chain end group titration showed only polycarbonate end groups and no residual PEO–a finding consistent with ABA block formation. DOSY NMR spectroscopy on the polymer samples showed a single diffusion coefficient, whereas mixtures of polymers showed two different diffusion coefficients. Block polymer composition (wt % PC) was determined by ^1^H NMR spectroscopy (Figure S5), and it was straightforward to modify the composition by changing the PEO loadings and reaction times (Table S2).

Polymers with different PEO mid-segment lengths (EO repeat units, *n*) and volume fractions of polycarbonate (*f*_*PC*_) referred as CEC(*n*, *f*_*PC*_) were synthesized to investigate the influences on the mechanical properties and ionic conductivity (Table 1). Very short PEO segments (*n* = 23 and 76), regardless of *f*_PC_ content (0.11–0.78), formed triblock polymers with unsuitable mechanical properties, whereas very long PEO blocks (*n* = 2272) yielded block polymers that were hard to process. Key samples in terms of optimized ionic conductivity, mechanical, and adhesive performance (*vide infra*) are **P1–P3** [Figure 1a(i)]. These contain *n* = 182 or 795 (*M*_n,PEO_ of 8 or 35 kg mol^–1^, respectively) and sufficient polycarbonate, *f*_*PC*_ > 0.25, to confer stability.

**Table 1:** Overview of Properites of Poly(carbonate-*b*-ethers) (CEC) Prepared

PEO^a^	*m*^b^	*f*_PC_^c^	*M*_n,SEC_ (kg mol^–1^)^d^	[*D̵*]^d^
***n*** = **795**	15	0.07	33.9	1.09
	40	0.16	40.9	1.11
	71	0.26	43.5	1.13
	98	0.33	49.0	1.11
	119	0.37	50.6	1.16
	247	0.55	67.3	1.21
	477	0.70	92.1	1.23
***n*** = **182**	27	0.37	12.7	1.06
	48	0.51	17.0	1.08
	113	0.70	20.4	1.07
***n*** = **76**	2	0.11	4.90	1.08
	15	0.43	6.78	1.14
	48	0.70	14.2	1.03
***n*** = **23**	4	0.40	1.90	1.06
	20	0.78	4.39	1.22
***n*** = **2272**	226	0.27	n/a	n/a

a EO repeat units.

b Total PC repeat units in triblock polymers.

c PC volume fraction (see Table S2 for calculation).

d Total CEC molar mass from SEC (vs PS standards, CHCl_3_ eluent). *D̵* = *M*_w_/*M*_n._

Radical-mediated thiol–ene reactions are high-yielding methods to introduce functional groups to polymer backbones.^27^ Phosphonic acid, that is, PO(OH)_2_ substituents, was attached to the polycarbonate blocks to tailor the adhesion/compatibility with the inorganic cathodes [Figure 1a(ii)]. These acids are established ligands for inorganic surface coordination, possess multiple binding modes, and are stabilized by the chelate effect. The efficiency of the post-functionalization was judged by ^1^H NMR spectroscopy of the purified polymers (precipitation from diethyl ether) by monitoring the reduction in the intensity of the alkene signals and the appearance of new alkylene signals consistent with those of 2-mercaptoethyl phosphonic acid (MEPA) attachment (Figure S6). It was observed that complete functionalization of the polycarbonate limited processability for composite cathode fabrication (Figure S7). Consequently, the PC block was partly functionalized (6 wt %) by controlling the stoichiometry of MEPA relative to the vinyl groups (see Figures S8–S10 for confirmation of partial functionalization by relative integration of ^1^H NMR signals and retention of polymer molar mass by SEC).

The solid polymers were processed into electrolyte (SPE) films by mixing with LiTFSI and using a solvent casting technique, under anhydrous conditions. The resulting stand-alone polymer films were dried under vacuum, at 70 °C, until no solvent residue was observed by NMR spectroscopy or thermogravimetric analysis (TGA) (Figure S11). Li ions can coordinate to both the carbonate groups and PEO oxygens. Subsquently, the amount of salt added was defined as the ratio of Li ions to EO plus carbonate (CO)-coordinating environments: *r* = [EO + CO]/[Li].

The PC and PEO blocks undergo microphase separation as indicated by two glass transition temperatures, one for the PEO (*T*_g_ = −46 to −37 °C) and the other for the PC microdomains (100–112 °C) (Figure 1b). Phase separation was also corroborated by small-angle X-ray scattering (SAXS) measurements (Figure S12). DSC of the films shows that adding LiTFSI disrupts the PEO crystallinity, as evidenced from the decrease in its melting point (Figure 1c); this finding is important as ionic conductivity is the greatest in the amorphous regions. One limitation of the PEO homopolymer is its semi-crystallinity (70–84%), which severely limits its RT ionic conductivity. For example, at *r* = 18 (22 wt % LiTFSI), the PEO crystallinity (χ_c_) for CEC(795,0.26) (**P1**) is roughly a third (χ_c_ 13%, *T*_m_ 42 °C) compared to that when no salt is present (χ_c_ 37%, *T*_m_ 47 °C). Adding more salt (*r* = 16) to the same sample is sufficient to yield completely amorphous PEO. The polymers show a wide operating temperature window (∼150 °C), as judged from the region between the lower and upper *T*_g_ (Figure S13). TGA indicated that the polymer electrolytes were stable to decomposition up to ∼230 °C (Figure S15).

FTIR spectroscopy supports Li-ion coordination to the carbonate carbonyl oxygen atoms, as indicated by a broadening of the carbonate C=O stretch and a shift to lower wavenumbers (1744–1739 cm^–1^) with increasing LiTFSI ratios (Figure 1d). ^7^Li NMR spectroscopy (CDCl_3_) indicates a preference for Li-ion coordination by the PEO chains at lower salt ratios (*r* = 13) (Figure S16). A single signal, at −0.5 ppm, was observed and was analogous to that observed for pure PEO/LITFSI. In contrast, for CEC(795,0.70) (**P2**) at higher salt ratios (*r* = 2, 66 wt %) and CEC(182,0.37) (**P3**), with shorter PEO segments, resonances were observed between pure PC (−0.7 ppm) and PEO (−0.5 ppm).

### Cathode Adhesion

**Figure 2:**
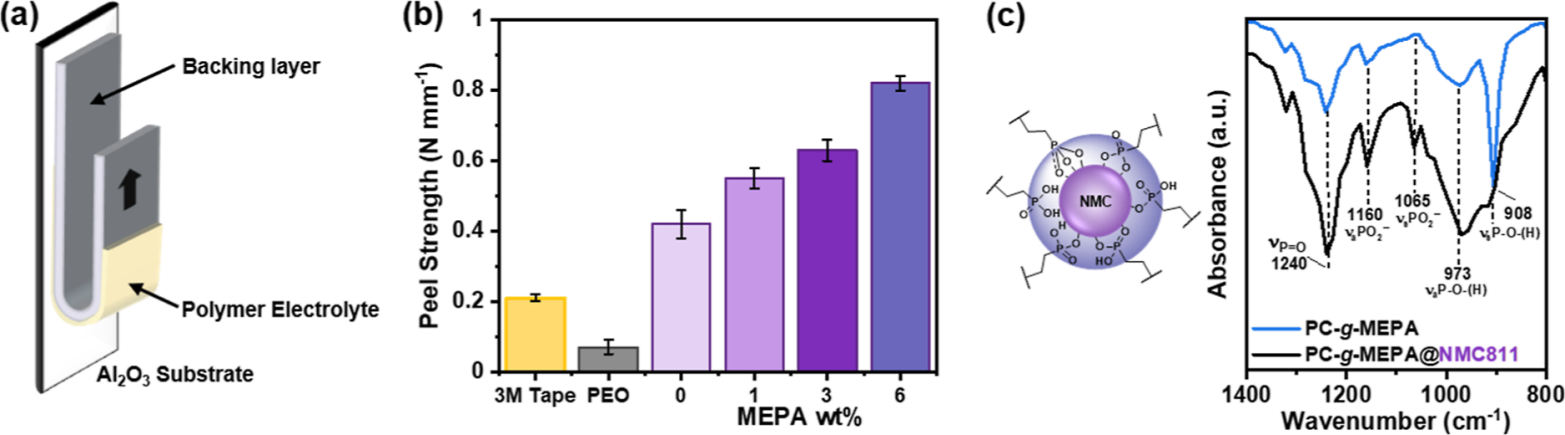
(a) Schematic of 180° peel test and (b) peel strength for **P1**/*r* = 13 as a function of wt % grafted phosphonic acid (MEPA). (c) Phosphonic acid binding modes and FTIR spectra zoomed into the regions for P-O stretching absorptions.

A pre-requisite for good interfacial adhesion is wetting of the polymer on the active cathode’s surface. For this to occur, the surface energy of the polymer should be less than that of the solid inorganic oxide. The surface energy of the polymer/LiTFSI films was determined using contact angle measurements and the application of Owens/Wendt theory (see Supporting Information for details). The results indicated that all the poly(carbonate-*b*-ether-*b*-carbonate) electrolytes should wet the cathode surface (Table S3). Subsequently, surface adhesion was investigated using 180° peel tests, conducted using alumina as a model substrate for the oxide cathode surface (Figure 2a). Polymer solutions, with differing wt % MEPA, were coated on alumina using a doctor blade (100 μm thickness). The force required to peel the polymer film from the oxide surface was measured and correlated to the peel strength (Figure S17). These results clearly illustrate the benefits of partial functionalization, with the 6 wt % MEPA sample showing 11 × greater peel strength than that of PEO (Figure 2b). The impacts of varying LiTFSI content, PEO mid-block length, and PC on adhesion were all investigated (Figure S17). Values of *n* > 76 and *f*_PC_ > 0.2 resulted in adhesive but processable films.

FTIR spectroscopy was used to probe the phosphonic acid coordination chemistry with the NMC cathode surface. In these experiments, pure PC featuring 100%-MEPA functionalization was mixed with NMC particles, and the FTIR spectrum of the coated particles was compared to that of the pure polymer (Figure 2c). In the P-O stretching region, from 900 to 1000 cm^–1^, the polymer-coordinated cathode shows a broad absorption, whereas the pure polymer (PC-*g*-MEPA) shows two absorptions attributed to asymmetric and symmetric P-O-(H) stretches. This change suggests that hydrogen bonding occurs on the surface, and a new absorption, at 1065 cm^–1^, is typical of PO_2_^–^, suggesting anionic surface coordination. The presence of the P=O stretching vibrations, at 2040 cm^–1^, which are absent for tridentate coordination, suggests that the phosphonic acids coordinate to NMC by a combination of hydrogen bonding and mono- and bi-dentate anionic binding modes.

### Ionic Conductivity

**Figure 3:**
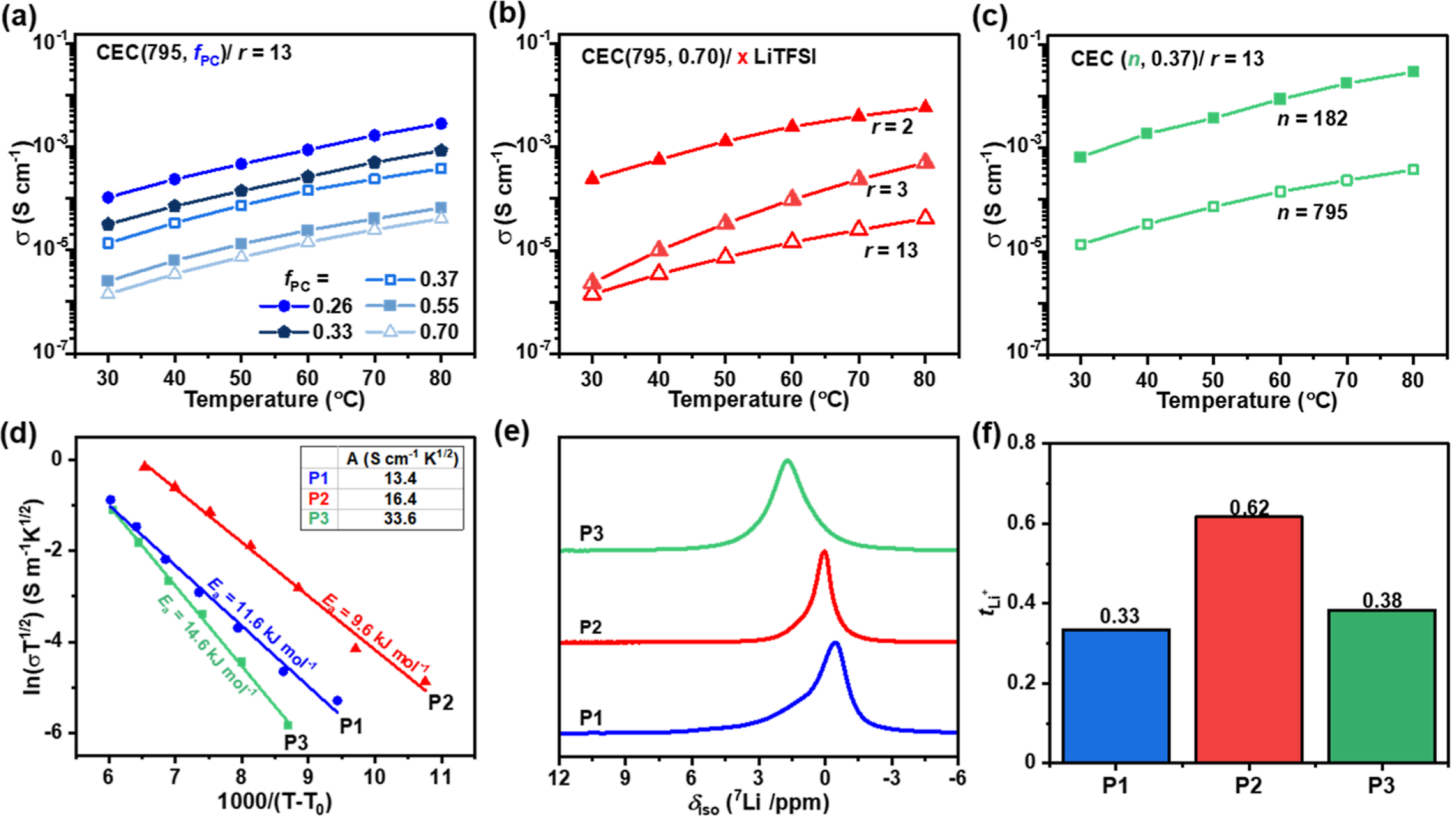
Ionic conductivity (σ) for SPE films as a function of temperature: (a) varying PC volume fraction (*f*_PC_) at a fixed PEO (*n* = 795) and salt ratio (*r* = 13). (b) Varying salt ratio for *n* = 795 and *f*_PC_ = 0.70. (c) Different PEO mid-segments (*n*) at *f*_PC_ = 0.37/*r* = 13. (d) VTF plots for **P1**/*r* = 13, **P2**/*r* = 2 and **P3**/*r* = 13 (*i.e.*, best from a–c). (e) Solid-state ^7^Li NMR. (f) Lithium transference numbers (*t*_Li+_).

Electrochemical impedance spectroscopy (EIS) was used to measure the ionic conductivities of the polymer electrolytes. Films were punched into discs with 90–130 μm thicknesses, as measured by digital microscopy (Table S4). Measurements were performed as a function of temperature (RT-80 °C) and salt content. First, at fixed PEO mid-segment length (*n* = 795), a series of polymer electrolytes differing in PC length (*f*_PC_ = 0.26–0.70) were investigated (Figure 3a). The salt ratio was kept constant at an optimized value of *r* = 13 (Figure S20). The ionic conductivity increased with decreasing *f*_PC_, which was attributed to the higher fraction of the conducting PEO phase and, presumably, to reduced polyether chain mobility due to the physical cross-linking/network formed by the outer, rigid PC blocks.^28^ Fortunately, the high ionic conductivity at low *f*_PC_ correlates with the polymer structures most likely to be elastomeric and hence best able to mitigate cathodic volume changes. It is already known that related ABA block polymers are thermoplastic elastomers at specific hard-domain volume fractions, *f*_PC_ < 0.30.^29^ Importantly, the room-temperature ionic conductivity of **P1**, *f*_PC_ = 0.26, is high at 1.1 × 10^–4^ S cm^–1^. This conductivity is an order of magnitude higher than that of PS-*b*-PEO/LiTFSI systems (1.2 × 10^–5^ S cm^–1^ at RT) and several orders greater than that of PEO (∼10^–7^ S cm^–1^ at RT).^28,30^ The increased ionic conductivity arises from both suppressed PEO crystallinity and from conductivity in the PC phase. It supports recent work showing that attaching a conductive block polymer to PEO improves its ionic conductivity.^31^

Another benefit is that the block polymers show higher oxidative stability with increasing carbonate content (vs Li^+^/Li, Figure S22). This parameter is important for any applications in a composite cathode. To optimize stability and conductivity, the triblock polymer with the highest stability, that is, *f*_PC_ = 0.70, was investigated at higher salt loadings (Figure 3b). At *r* = 2, this polymer achieved a RT conductivity of 2.3 × 10^–4^ S cm^–1^. It also showed a high lithium transference number, tensile toughness, and stability against LPSCl (*vide infra*). Finally, the impact of changing PEO length was probed: at short lengths (*n* = 182 vs 795), higher conductivities were observed at comparable *f*_PC_. At *n* = 182, *f*_PC_ ∼ 0.37 was required for mechanical integrity (Figure 3c).

The temperature dependence of the lithium-ion conductivity was expected to follow the Vogel–Tamman–Fulcher (VTF) model
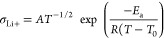
where *T*_0_ = *T*_g_–50 K and parameters *A* (relating to the free charge carrier concentration) and *E*_a_ (activation energy for ion transport) are obtained from linear fits. The PC-PEO-PC triblock polymers showed the following trends: (1) Increasing *f*_PC_ or MEPA content (*i.e.*, physical cross-linking) had little impact on *E*_a_ but a larger influence on *A*. (2) Increasing salt content and *n* influenced both *E*_a_ and *A* (Figure S23). VTF plots are shown for the three most conductive polymers **P1**–**P3** (Figure 3d). The extracted *E*_a_ and *A* values rationalize the trend in ionic conductivity for the lead polymers: **P3** > **P2** > **P1**. Specifically, **P3** has the highest concentration of free charge carriers (*A*), and **P2** has the lowest activation energy for ion transport. Overall, the *E*_a_ values (9.6–14.6 kJ mol^–1^) are comparable with those of other electrolytes comprising lithium salts in amorphous PEO.^32^

Solid-state ^7^Li NMR spectra, for **P1–P3**, show increasingly higher frequency shifts following the same order as the conductivity data (Figure 3e). The shift coincides with a greater contribution from PC-Li environments and, presumably, increased free-ion movement within **P3**, consistent with the higher *A* value. Pulsed-field-gradient NMR spectroscopy of the solid-state polymers, at 60 °C, allowed for estimation of the diffusion rates of the ^7^Li- and ^19^F-containing species (Figure S24). If complete ion dissociation is assumed, this roughly correlates not only to diffusion of the Li ions and TFSI anions but may also contain contributions from neutral ion pairs and charged ion clusters. Accordingly, **P3** showed the slowest Li-ion diffusivity and diffusion rates decreased in the order: **P2** > **P1** > **P3** (Table S5), reflecting the trend in activation energies. Next, the lithium-ion transference number (*t*_Li+_), or contribution of Li ions to the total conductivity, was estimated using this data for the mobility of the cations and anions (a value approaching unity being desirable). **P2** showed the highest transference number of 0.62, consistent with its high salt loading (Figure 3f). Polycarbonates are generally reported to have higher permittivity and weaker coordination to Li ions than polyethers, which could rationalize the higher *t*_Li_ observed with more PC (**P3** > **P1**) and compared to pure PEO (*t*_Li_ < 0.2). Hydrogen bonding (*i.e.*, *via* the MEPA) to the anion may also retard anion migration, increasing *t*_Li+_ for the triblock polymers.^33^ Interestingly, **P1–P3** block copolymers show higher conductivities than random copoly(carbonate-*ran*-ethers) reported previously (entries 6–10, Table 2). It may be because the phase-separated morphologies, in the block polymers, provide channels that facilitate ion movement.

**Table 2:** Summary of Electrochemical and Mechanical Data

			σ (mS cm^–1^)^c^			
entry	Polymer Electrolyte^a^	*T*_g_ (°C)^b^	30 °C	60 °C	*G*′ (MPa)^d^	*t*_Li+_^e^
1	**P1**	–40, 105	0.11	0.34	0.82	0.33
2	**P2**	–23, 90	0.23	2.5	67	0.62
3	**P3**	–45, 84	0.67	9.1	0.52	0.38
4^30a^	PEO	–64	∼10^–4^	0.14	0.4–1^f^	0.2
5^28,30b^	PEO-PS	–40, 80	0.012	0.23	10	0.1
6^24e^	PEO_34_-PC	–48	0.037	∼0.1	n/a	n.a
7^24f^	PEO_34_-PC-*X*	–45	0.032	1.3	<0.01	0.59
8^24b^	PEEC	–34	0.016	∼0.1	n/a	0.40
9^24a^	P(EC-co-EO)	–43	∼0.1	0.48	n/a	0.66
10^24c^	PTEC	–36	0.011	0.2^g^	n/a	0.39

a Salt content varies; parameters reported for electrolytes at their optimized salt ratio for ionic conductivity. **P1–P3** = this work, PS = polystyrene, PEO34-PC = poly(ethylene oxide carbonates) with 34 EO units to every carbonate, PEO_34_-PC-*X* = cross-linked with 10 wt % MA, cross-linked PEEC = poly(ethylene ether carbonate), and PTEC = poly(triethylene glycol carbonate).

b Glass transitions from DSC.

c Ionic conductivity.

d Storage modulus at 30 °C.

e Lithium transference number. n/a = not reported.

f Dependent on crystallinity.

g *T* = 80 °C.

**Figure 4:**
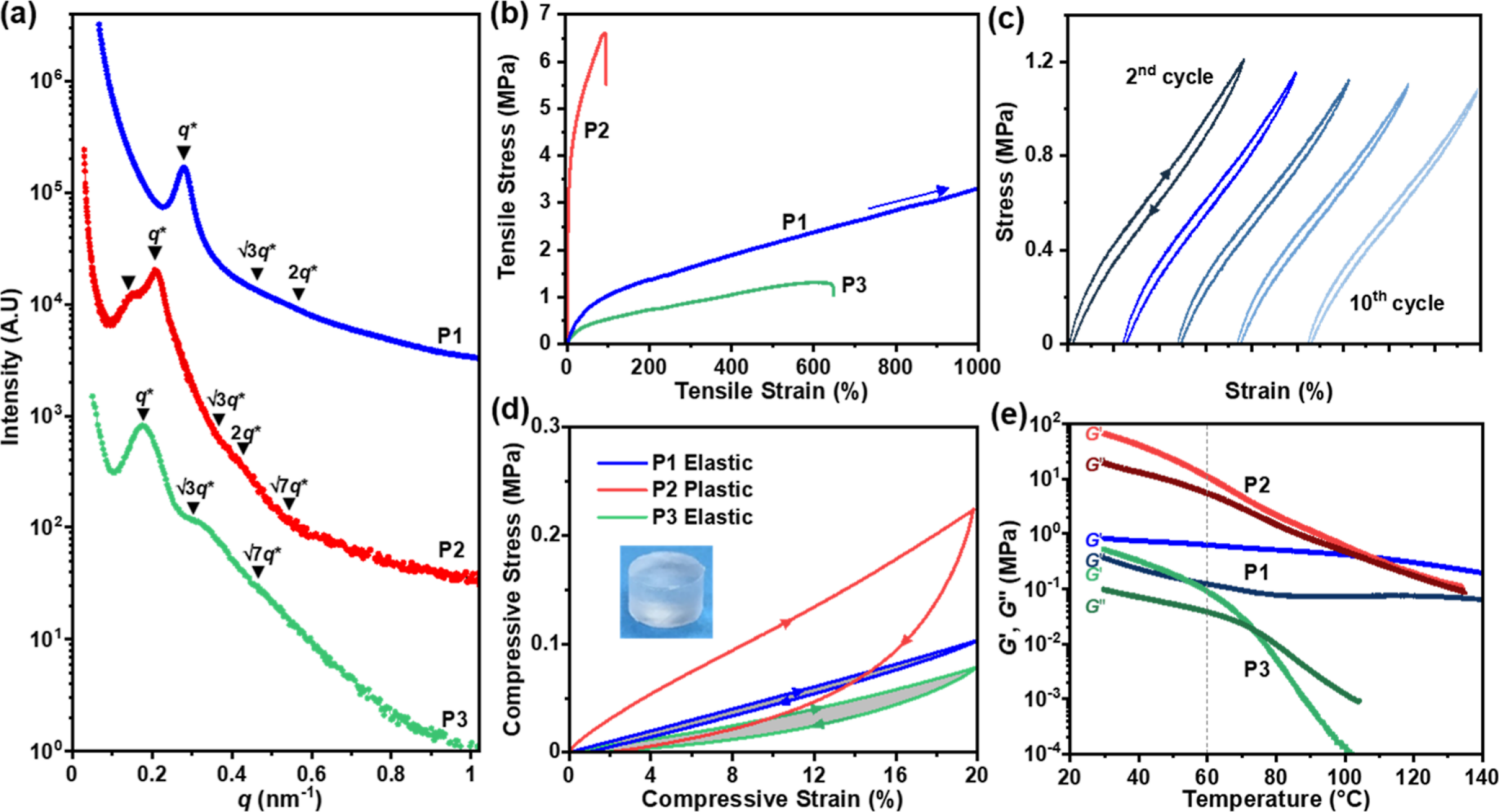
(a) RT SAXS profiles (vertically shifted for comparision). (b) Tensile stress–strain data for **P1–P3** (10 mm min^–1^ strain rate). (c) Cyclic tensile testing of **P**_**1**_ to 200% strain (horizontally shifted). (d) Tensile compressive properties (1 mm min^–1^ strain rate). (e) Rheological measurements of storage (*G*′) and loss (*G*″) moduli (2 °C min^–1^, ω = 1 Hz).

SAXS measurements of the polymer electrolyte films (measured at RT and without annealing) suggest that there is some long-range ordering in all the samples (Figure 4a). **P1** and **P3** show scattering peaks consistent with hexagonally packed cylinders or spherical morphologies of PC in a PEO matrix. The domain spacings (*D*), based on the principal scattering peaks (q*), are 24 and 36 nm, respectively. **P2** has a mixed morphology with a domain spacing of ∼38 nm. In amorphous phase-separated block polymers, the full width at half maximum (FWHM) of q* can be approximated to the average sizes of these ordered regions (*G*): *G* ∼ 1/FWHM based on the Scherrer equation. Smaller grain sizes are associated with higher ionic conductivities.^34^ Accordingly, it is inferred that **P1**, with the lowest conductivity, has grain sizes ∼1.6 × those for **P2** and **P3** (Figure S25).

### Mechanical Properties

Few studies of polymers used in composite cathodes take into consideration their mechanical features, and as a result, quantifying the most desirable properties is difficult.^15a^ To investigate the mechanical property–performance correlations for these block polymers, the behavior of the SPEs under tension was investigated using dumbbell specimens, cut from the films according to specimen type-5B ISO standard 527-2. All samples were stored in a glovebox prior to testing to minimize the influence of water on the mechanical properties. The Young’s modulus (*E*_y_) was determined from the initial linear stress–strain region (0.025–0.25% strain) (Figure 4b for **P1–P3** and Figures S26–S28 for other polymers and variable salt contents). Both **P1** and **P3** show a typical elastomer behavior with linear stress and strain relationships. **P1**, in particular, shows an excellent elastic recovery of 98.3 ± 0.2% at 200% strain, a high resilience of 91.6 ± 0.9% (*i.e.*, low hysteresis), and minimal residual strain (3.5 ± 0.5%) (Figure 4c). High elasticity was proposed as important in the composite cathode to buffer volume changes. Tensile toughness increases with salt content for **P2** (Figure S26).

Next, the polymers’ behavior under compression was investigated to mimic the forces present during contraction of NMC811 on delithiation (Figure 4d). Both **P1** and **P3**, under a compressive strain of 20%, show good elastic strain recovery, whereas **P2** behaves as a plastic. Batteries are required to operate over a range of temperatures, and thus, understanding the variation in mechanical properties with temperature is important. Shear rheology experiments were performed to characterize the storage (*G*′) and loss (*G*″) moduli from 30 to 140 °C in the linear viscoelastic region (Figure 4e). At the targeted cell operating temperatures of 30–60 °C, **P1–P3** all behave as elastic solids (*G*′ > *G*″). In this range, *G*′ is greatest for **P2** due to its high PC content and **P1** exhibits a classic elastomer behavior, where *G*′ is temperature-independent. **P3** shows a *G*′/*G*″ cross-over at 74 °C, above which the polymer electrolyte exhibits a “liquid-like” behavior. Using time–temperature superpositions (following WLF theory), master curves of *G*′ and *G*″ as a function of frequency were generated (Figure S30). **P1** showed only a rubbery plateau region (*G*′ > *G*″) and no modulus cross-overs, but **P2** and **P3** showed some viscous behavior at low frequency, where *G*″ > *G*′. To quantify, the flow transition relaxation time (τ_f_) was determined from the reciprocal frequency at the *G*′/*G*″ cross-over. Values of 4.4 s for **P2** and 57 s for **P3** (at 60 °C) indicate that these SPE should relax and “flow” at shorter timescales. Despite this, **P1–P3** all show low creep rates under an applied compressive stress of 1 MPa (10^–4^/10^–5^ % s^–1^, Figure S31). We posit that these mechanical characteristics are suitable for the proposed application as volume change buffers in a composite cathode.

### Battery Performance

**Figure 5:**
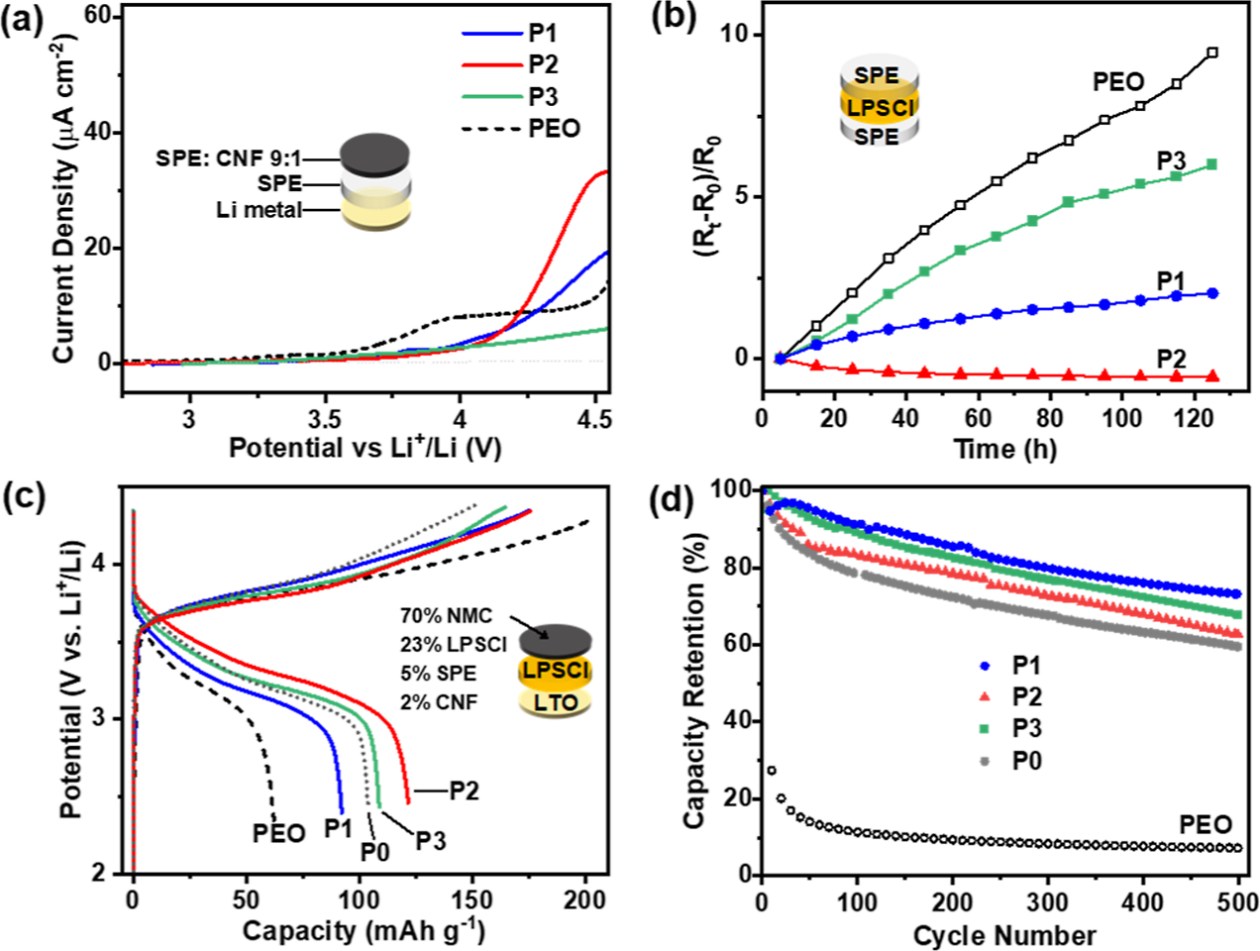
(a) Oxidative stability: LSV from open-circuit voltage to 6 V at 0.05 mV s^–1^, 60 °C, and 10 MPa; working electrode = polymer electrolyte (SPE)/CNF composite. (b) Stability vs LPSCl: change in resistance with time. *R*(*t*) = resistance at time, *t*, measured by EIS (RT) after 10 h time intervals at 60 °C; *R*(0) = initial resistance (Figure S33). (c) First charge–discharge voltage profiles at 0.5 C (1.75 mA cm^–2^), 60 °C, 1 MPa stack pressure. NMC811 active material = 15 mg cm^–2^. (d) Capacity retention vs cycle number. See Figure S34 for Coulombic efficiency and rate capability.

The cell performance of the three lead polymers was investigated: **P1** is a high-performance thermoplastic elastomer (elastic recovery >98%), **P2** is a polymer-in-salt composition with high *G*′, and **P3** is a soft elastomer (elastic recovery 80.7 ± 0.4%). Prior to cell fabrication, the polymers’ oxidative stability was evaluated by linear sweep voltammetry (LSV), at a slow scan rate of 0.05 mV s^–1^ (Figure 5a). In this experiment, the working electrode was a composite of the polymer with carbon nanofibers (CNFs).^35^ A polymer electrolyte layer was then stacked against a lithium metal counter electrode. Under these conditions, oxidative stability decreased in the order: **P3** > **P2** > **P1**. The sequence reflects the reduced number of EO units (**P3**) and increased PC block lengths (**P2**). Importantly, all the polymers are stable up to and above 4 V and outperform the PEO homopolymer (<3.5 V).

Janek *et al.* recently demonstrated an interfacial reaction between PEO electrolytes and solid-state electrolyte, LPSCl, yielding polysulfide degradation products.^36^ To assess the chemical stability of the triblock polymer electrolytes versus LPSCl, interfacial resistance was measured of polymer electrolyte films sandwiched between LPSCl. The cell was heated at 60 °C, and at regular 10 h intervals, RT impedance measurements were recorded. It was found that using lithium bis(fluorosulfonyl)imide (LiFSI), over LiTFSI, resulted in favorable lower interfacial resistance values, and subsequently, this salt was used for cell testing (Figure S32). Ionic conductivity and mechanical performance of **P1–P3** were not significantly influenced by the smaller anion (Table S6).The control polymer, PEO, shows increasing resistance with time and the formation of additional interfaces, indicating chemical reactivity and degradation of LPSCl. In contrast, for **P2**, the resistance initially decreases before plateauing, which may suggest the formation of a beneficial interphase. After 5 days, little difference in conductivity behavior was observed, indicating that the polymer electrolyte shows good chemical stability against LPSCl (Figures 5b and S33). This stability might be attributed to the beneficial properties of both high lithium salt content and the stabilization afforded by the polycarbonate block. At lower salt loadings, **P1** and **P3** show less effective stability than that of **P2**, but compared with PEO, they showed a slower rate of change to the interfacial resistance.

Next, composite cathodes were prepared with **P1, P2,** or **P3** using CNFs for electrical conductivity, LiNbO_3_-coated polycrystalline NMC811 cathode material, and LPSCl ceramic electrolyte. The powders were homogeneously mixed, in a glovebox, by grinding, with a pestle and mortar, and cold-pressed to form a pellet (400 MPa). An LTO-based composite electrode served as the counter electrode, and lithium metal was used as the reference electrode. The cells were charged–discharged at 60 °C, 1 MPa stack pressure, 0.5 C rate (1.75 mA cm^–2^). Initial discharge capacity measurements show that **P2** and **P3** have higher capacities than cells without any polymer (**P0**), PEO, or **P1** (Figure 5c). This result is consistent with their higher conductivities compared with **P1** and their good interfacial properties after cell densification. Over multiple charge–discharge cycles, all the polymers showed promising performances (Figure 5d). Capacity retention, determined as the discharge capacity at cycle *x* relative to the initial discharge capacity, represents the long-term battery stability. The highest-performing elastomer, **P1** (98% strain recovery and 92% resilence), showed the highest capacity retention (86% after 200 cycles) compared to softer less-resilient elastomer **P3** (83%) and more plastic **P2** (79%). Importantly, all samples containing these polymers were superior to the cell fabricated without any polymer (73%). As a result of the capacity loss for the no polymer setup, after 100 cycles, **P1** retains a higher discharge capacity (Figure S34). After 500 cycles, **P1** shows a 23% higher capacity retention compared to the no polymer control, whereas **P2** and **P3** showed 5 and 14% greater capacity retention, respectively. This indicates that the elastomeric behavior is more crucial for accommodating volume changes than **P2** and **P3,** which showed viscous flow characteristics. In future, it should be feasible to increase the electrochemical stability of **P1** and further enhance its ionic conductivity. Cells fabricated using only the PEO homopolymer showed poor capacity retention. This latter finding highlights the benefits of these block polymers and likely arises from its lower oxidative stability and liquid state. Improvements in capacity and capacity retention were also observed when comparing solid-state composites prepared using **P1–P3** with traditional nitrile butadiene rubber (NBR) or poly(vinylidene fluoride) (PVDF) binders (Figure S35). The enhanced interfacial adhesion afforded by the phosphonic acids in **P1** compared to non-polar NBR resulted in a 23% improvement in capacity retention over 500 cycles. Discharge capacities were also greater due to the ionic conductivity of **P1–P3** over the non-conductive binders.

## Conclusions

In summary, a series of new triblock polymers, poly(carbonate-ether-carbonate), showed real promise as conductive, mechanically robust, stable binders in composite cathodes used in solid-state lithium-ion batteries. The block polymers featured amorphous polyethylene oxide (PEO) mid-segments and rigid polycarbonate outer blocks. The polymers were straightforwardly prepared using PEO macroinitiators and CO_2_/epoxide ring-opening copolymerization (ROCOP) on a 10–15 g scale. They all featured controllable phosphonic acid functionality, as a side chain substituent, which improved interfacial adhesion with cathode particles. For the same PEO mid-segment length (795 EO units) and lithium salt ratio (1 Li ion: 13 carbonate plus EO coordinating environments, *r* = 13), lower volume fractions of polycarbonate (*f*_PC_ < 0.3) yield elastomeric electrolytes with higher ionic conductivity (10^–4^ S cm^–1^ at 30–60 °C). Shorter PEO mid-segments (182 EO units) resulted in block polymers showing increased conductivity (9.1 × 10^–3^ S cm^–1^ at 60 °C) and yielded soft elastomers (*f*_PC_ = 0.37) with high oxidative stability (>4 V). A block polymer with a high lithium salt content (*r* = 2) and high PC block lengths (*f_PC_* = 0.70) also showed high ionic conductivity (2.5 × 10^–3^ S cm^–1^ at 60 °C) and impressive stability versus LPSCl solid electrolyte and lithium transference numbers >0.6. These leading PC-*b*-PEO-*b*-PC electrolytes all showed higher conductivity, transference numbers, and oxidative stability compared to pure PEO systems, random EO/PC copolymers, or other PEO-based block copolymers. The enhanced performances are attributed to suppression of PEO crystallinity, improved oxidative stability afforded by the polycarbonates, and microphase separation into hexagonally packing cylinders and spherical morphologies with diffused phase boundaries accelerating ion transport.

The polymer electrolytes were applied in composite cathodes comprising polycrystalline NMC, LPSCl, and carbon–the composites showed better capacity retention than equivalent cells fabricated without any polymer or using the pure PEO, NBR or PVDF polymers as controls. The triblock polymer with the best elastomeric and adhesive performance showed the most improved capacity retention (86% over 200 cycles) despite having somewhat lower conductivity and (electro)chemical stability compared with the other polymers. This investigation provides a proof of concept for the potential of block polymers in SSBs. In future, the polymers should be investigated with different cell components and other (multi)block polymer structures should be targeted.

## Supporting Information Available

Detailed experimental procedures for polymerizations and polymer characterization data (NMR, SEC, TGA, additional DSC, mechanical, and ionic conductivity data) (PDF)
