# Supplementary material for: Buffering Volume Change in Solid-State Battery Composite Cathodes with CO2-Derived Block Polycarbonate Ethers
Source: J Am Chem Soc. 2022 Sep 19;144(38):17477–86. doi: 10.1021/jacs.2c06138 (PMC9523710; doi:10.1021/jacs.2c06138)
Supplement: Supplementary file 1 — ja2c06138_si_001.pdf [file ja2c06138_si_001.pdf]

Buffering Volume Change in Solid State Battery Composite Cathodes with CO_2-derived *Block* Polycarbonate-ethers

Georgina. L. Gregory,[a*] Hui Gao,[b] Boyang Liu,[b] Gregory J. Rees,[b] Xiangwen Gao,[b] Mauro Pasta,[b*] Peter Bruce,[b*] Charlotte K. Williams[a*]

[a] Chemistry Research Laboratory, University of Oxford, 12 Mansfield Road, Oxford, OX1 3TA, UK
[b] Department of Materials, University of Oxford, Parks Road, Oxford, OX1 3PH (UK)

*Email: georgina.gregory@chem.ox.ac.uk; charlotte.williams@chem.ox.ac.uk; mauro.pasta@materials.ox.ac.uk; peter.bruce@materials.ox.ac.uk

Table of Contents

Materials 1
Methods 1
Experimental Procedures 2
Fig. S1 Ring-Opening Copolymerization (ROCOP) mechanism 4
Table S1. Details of PEO macroinitiators used in this work. 5
Table S2. Synthesis of PC-*b*-PEO-*b*-PC (CEC) triblock polymers. 5
Fig. S2 Size Exclusion Chromatography (SEC) 6
Fig. S3 Polymer end-group analysis by phosphorous test. 6
Fig. S4 DOSY NMR for CEC(182,0.51). 7
Fig. S5 Representative NMR spectra of purified PC-b-PEO-b-PC 8
Fig. S6 NMR analysis of MEPA post-functionalized PC-PEO-PC triblock 9
Fig. S7 Gel behaviour observed with higher MEPA content (wt%). 9
Fig. S8 ^{1}H NMR spectra ($CDCl_3$) confirming partial MEPA functionalization of PC. 10
Fig. S9 SEC ($CHCl_3$ eluent) of CEC(795,0.55)-*g*-MEPA (3 wt%). 10
Fig. S10 Additional characterization of partially MEPA decorated polymers. 11
Fig. S11 Picture of SPE film 11
Fig. S12 Additional SAXS data 11
Fig. S13 Dynamic Mechanically Thermal Analysis (DMTA) conducted for P1/ *r* = 13. 12
Fig. S14 Additional DSC data for polymers and polymer electrolytes. 12
Fig. S15 TGA curves. 13
Fig. S16 ^{7}Li NMR($CDCl_3$) of polymer electrolytes.. 13
Table S3. Surface energy measurements 13
Fig. S17 Additional 180° peel tests on alumina 14
Fig. S18 SEM image of polymer-NMC composite 14
Fig. S19 180° Peel tests of composite cathode on Al current collector.. 15
Table S4. SPE film thickness measurements 15
Fig. S20 Optimisation of LiTFSI content for CEC(795,0.37). 16
Fig. S21 Li-Ion conductivity as a function of MEPA wt%/ *r* = 13 17
Fig. S22 Oxidative stability as a function of f_{PC}. 17
Fig. S23 Additional VTF Plots 17
Fig. S24 Diffusion PFG Decays 18
Table S5. Diffusion PFG-NMR 18
Fig. S25 Plot of 1/FWHM from SAXS (*q) vs ionic conductivity for P1, P2, P3.** 18
Fig. S26 Toughening of P2 with salt ratio. 18
Fig. S27 Additional stress-strain curves for P1. 19
Fig. S28 Mechanical properties as a function of PC content. 19
Fig. S29 Additional elastic recovery data 20
Fig. S30 Time-Temperature Superposition (TTS) master curves 21
Fig. S31 Creep experiments 22
Fig. S32 Interfacial resistance for P2 with (a) LiTFSI and (b) LiFSI 22
Fig. S33 P2/LPSCl Interfacial resistance. 22
Table S6. Ionic conductivity and mechanical parameters of P1-P3 with LiFSI 23
Fig. S34 Additional cell performance data 23
Fig. S35 Cell performance compared to traditional binders 24
References 24

Materials

4-vinyl cyclohexene oxide, vCHO was purchased from ACROS and purified by drying over CaH_2 and fractional distillation. Poly(ethylene oxide) (PEO) was purchased from Sigma Aldrich and dried under vacuum at 110 °C for 24 h immediately prior to use. Anhydrous diethyl carbonate (DEC) was purchased from Sigma Aldrich, degassed by freeze-pump thaw (3 cycles), and stored over 3 Å molecular sieves under nitrogen. Lithium bis(trifluoromethanesulfonyl)imide (LiTFSI) was purchased from Sigma Aldrich and dried under vacuum at 110 °C for 48 h before being stored in a glovebox. Lithium bis(fluorosulfonyl)imide (LiFSI) was purchased from Solvionic and stored in a glovebox prior to use. CP Grade (BOC, 99.995 %) CO_2 was used for all polymerizations and dried through two VICI purifier columns. Anhydrous THF for polymer electrolyte film formation was obtained from a Solvent Purification System (SPS), degassed by freeze-pump thaw (3 cycles) and stored in a glovebox over 3 Å molecular sieves.

Methods

NMR: ^{1}H, ^{7}Li, ^{31}P {^{1}H} and ^{13}C{^{1}H} NMR were recorded on a Bruker Avance III HD 400 MHz spectrometer. DOSY spectra were recorded on Bruker Avance III HD 500 MHz spectrometer.

Pulsed field gradient stimulated echo (PFGSTE) NMR: All pulsed field gradient (PFG) nuclear magnetic resonance (NMR) measurements were completed at 9.45 T ($\upsilon_0(^{19}F)$ = 376.58, and $\upsilon_0(^{7}Li)$ = 155.53 MHz) on Bruker Avance III HD spectrometer using a 5 mm single-axis diffusion probe with exchangeable ^{19}F and ^{7}Li ceramic heads. A stimulated echo pulse sequence was utilized with an effective gradient pulse duration (δ) of between 1-2 ms, and a diffusion time (Δ) of 20 ms, with the gradient amplitude varying between 0.1 and 15 T/m. The experimental temperature was stabilized at 333.1 K, and a 5 second recycle delay was used throughout. All data is fitted to $f(x) = I_0 \times e^{-\gamma^2 g^2 \delta^2 \frac{(\Delta - \delta)}{3} D}$, where for ^{19}F; δ = 1 ms, Δ = 20 ms, and γ = 25172 rad/sG, and for ^{7}Li; δ = 1 ms, Δ = 20 ms, and γ = 10397 rad/sG. A R^2 of 0.999, errors of <1% (<1x10^{-13} m^{2}s^{-1}) and normal distributions of the residuals were observed for all experiments.

Size Elusion Chromatography (SEC): Polymer (2-10 mg) dissolved in HPLC grade $CHCl_3$ (1 mL) was syringe filtered through 2 µm filters before being injected into an Agilent PL GPC-50 instrument, with two PSS SDV 5 µm linear M columns heated to 30 °C. HPLC grade $CHCl_3$ was used as the eluent at a flow rate of 1.0 mL min^{-1} with RI detection calibrated using a series of narrow molecular weight polystyrene standards. Agilent SEC post-run program was used to analyze the data.

Differential Scanning Calorimetry (DSC): These were recorded for purified polymer samples and solid polymer electrolyte films on a Mettler Toledo DSC3 Star calorimeter under a nitrogen flow (80 mL min^{-1}). Samples were heated to 200 °C and held for 5 minutes to remove any thermal history before heating and cooling from -80 to 200 °C at a rate of 10 °C min^{-1}. Glass transition temperatures (T_g) were determined from the midpoint of the transition in the second heating curve.

Thermogravimetric Analysis (TGA): Measured on Mettler-Toledo Ltd TGA/DSC 1 system. Powder polymer samples were heated from 30 to 500 °C at a rate of 5 °C min^{-1}, under N_2 flow (100 mL min^{-1}).

Phosphorus end group tests: Following a literature procedure,[1] to polymer (40 mg) dissolved in $CDCl_3$ (0.4 mL) was added 40 µL of solution containing Cr(acac)$_3$ (5.5 mg) and internal standard, bisphenol A (400 mg) in pyridine (10 mL) followed by 40 µL of 2-chloro-4,4,5,5-tetramethyl dioxaphospholane.

Rheology: Shear storage (*G*') and loss moduli (*G*'') were measured on a TA instruments Q800 with 25 mm stainless steel platens. Measurements were conducted in the linear viscoelastic region as determined by an amplitude sweep conducted at 30 and 200 °C. The polymer electrolyte was heated from 30 to 200 °C at 2 °C min^{-1}, 1 Hz frequency, 0.1% amplitude strain. Frequency sweeps were also conducted between 30 and 100 °C at 10 °C intervals.

Dynamic Mechanical Thermal Analysis (DMTA): Storage (*E*') and loss moduli (*E*'') were measured on a TA instruments Q850 in tension mode at 0.1% strain, 1 Hz frequency from -60 to +180 °C at a heating rate of 3 °C min^{-1}.

ATR-FTIR: IR spectra of thin films were recorded on a Perkin Elmer Spectrum 100 (FT-IR) with an AT-IR crystal or a Varian FT-IR 3100 spectrophotometer (Golden Gate). All the IR measurements were performed in the reflection mode at a resolution of 4 cm^{-1}.

Tensile Testing: Dumbbell specimens were cut from polymer electrolyte films according to ISO 527-2, specimen type 5B with Zwick ZCP020 cutting press (length= 35 mm, gauge length = 10 mm, width = 2 mm). Monotonic uniaxial extension experiments were carried out on a Shimadzu EZ-LZ Universal testing instrument at an extension rate of 10 mm min^{-1}. An external camera was used to calculate the Young's Modulus, E_y within the 0.025-0.25% strain region. 10 Specimens were tested for each material. Cyclic tensile tests were conducted to 20 or 200% strain at a rate of 10 mm min^{-1}. 10 Cycles were measured for each specimen, 3 specimens for each sample.
Compression Testing: Polymer electrolytes were pressed into pellets (10 mm diameter, 2 cm thick) using a Carver Hotpress at 80 °C and a 10 mm diameter die set. Clear, colourless pellets were inspected to ensure they were free from air bubbles prior to testing. Compression experiments were carried out on a Shimadzu EZ-LZ Universal testing instrument fit with compression jigs at a rate of 1 mm min^{-1}.
180° Peel Tests: Solid polymer electrolyte (0.4 g) in THF (4 ml) was cast using a doctor blade (80 µm wet film thickness) onto alumina sheets (20 × 80 mm), and the solvent was allowed to evaporate at RT. The coated alumina sheets were then dried in a vacuum oven for 72 h before testing. 3M® scotch tape was applied to the polymer electrolyte coated alumina. A corner of the coating was manually de-bonded from the alumina substrate, and a Shimadzu EZ-LZ universal tensile tester was used to peel the coating off at a 180° angle and rate of 305 mm/min. The force required to remove the coating was measured. Peel strength (N mm^{-1}) = Peel force/width.

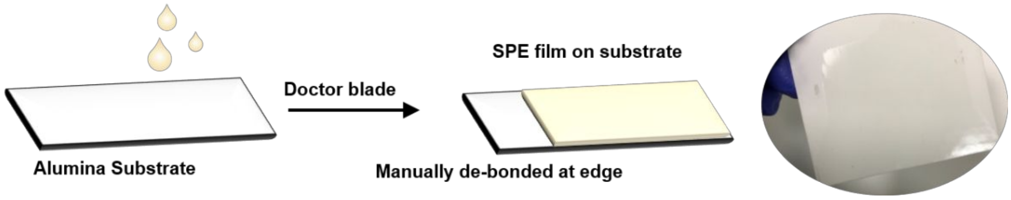

Electrochemical Impedance Spectroscopy (EIS): Discs of polymer electrolyte films were cut using a cutting press (16 or 18 mm diameter). The SPE discs were then sandwiched between two gold blocking electrodes (Au|SPE|Au) and placed inside a CESH cell. Impedance measurements were conducted between 0.1 MHz and 1 Hz using a Biologic Impedance Analyzer over 30 to 80 °C with 0.5 h soak time at each temperature. The resulting Nyquist curves were analyzed using EC-labs software to fit an equivalence circuit and determine resistance, R. Conductivity (σ) was then determined as: $\sigma = l/AR$, where A = the polymer electrolyte disc area and l = the electrolyte thickness (0.1-0.45 mm, Table S4).
Linear Sweep Voltammetry (LSV): These were recorded for solid polymer electrolyte discs (as described in EIS measurements above). Initial measurements were recorded at RT vs lithium foil in a PEEK cell with stainless steel (SS) counter-electrode (Li|SPE|SS). The open-circuit voltage (OCV) was first recorded for 12 hours to ensure stability before conducting experiments at 1 mV s^{-1} from the OCV to 5 V. For lead polymers **P1-P3**, LSV was also measured vs lithium metal counter electrode with a polymer-carbon nanofibre composite working electrode at 60 °C and 0.05 mV s^{-1} from the OCV to 6 V using Ni current collectors.[2]
Small Angle X-ray Scattering (SAXS): Polymer films were submitted to Harwell Diamond Light Source in a solid sample grid for SAXS analysis (DL-SAXS, P38 instrument). Scans (3 × 5 min) were conducted at camera lengths of 4.5 and 1 m, beam energy = 9.2 keV (using the Ga MetalJet). SAXS curves reported are an average of the 3 scans measured from data collected at 1 m. Experiments were conducted at RT on samples prepared to mimic processing conditions.

Experimental Procedures

General Polymerization Procedure: PEO with M_n = 35 kg mol^{-1} (9.3 g, 0.27 mmol, 20 equiv.) was dried under vacuum at 110 °C for 24 h to remove any adventitious water acting as a chain-transfer agent. At room temperature in an inert atmosphere, vCHO (10 ml, 80 mmol, 6000 equiv.) and $LMgCo(OAc)_2$ (10 mg, 0.013 mmol, 1 equiv.) were added. DEC (20 ml) was then added to the reaction mixture, and any remaining catalyst residue was rinsed into the solution. The nitrogen headspace was then replaced with CO_2 by 3 vacuum- CO_2 cycles before placing the solution in an oil bath preheated to 100 °C. Aliquots were taken from the reaction mixture at various time points under a stream of CO_2 gas and analyzed by ^1H NMR spectroscopy to monitor the conversion of vCHO monomer (3.1-3.2 ppm) to polycarbonate (4.76 ppm). For f_{PC} ~ 0.26, after 6 h, the reaction was cooled to RT and quenched

by adding benzoic acid (~16 mg, 0.13 mmol, 10 equiv.) dissolved in the minimum amount of dichloromethane. The polymer was isolated by precipitation on pouring the solution into a large excess of diethyl ether or methanol (~ 400 ml) and vacuum filtration. The polymer was further purified by rinsing the filtrate with ether/methanol and toluene. These precipitations and washings were repeated ×3 to obtain a pure white powder (12.5 g, 90% yield). Without these purification steps, a theoretical maximum of ~8.7 ppm metal residue (Mg plus Co) would be expected to remain in the polymer based on 10 mg of catalyst producing 12.5 g of polymer. NB. for f_{PC} > 0.5, methanol should be used as the antisolvent and the product not rinsed with toluene; for $M_{n,PEO} \leq 8$ kg mol^{-1}, hexane should be used to precipitate the polymer.

General Procedure for Thiol-ene Reaction: To triblock polymer ($M_{n,PEO}$ = 35 kg mol^{-1}, f_{PC} =0.26) (1 g, 1.6 mmol C=C, 1 equiv. C=C) dissolved in degassed THF (10 ml), MEPA (66 mg, 0.46 mmol, 0.3 equiv.) followed by DMPA (12 mg, 0.046 mmol, 0.03 equiv.) was added. NB. for polymers with f_{PC} < 0.3, gentle heating is required to disrupt PEO crystallinity and dissolve the block polymer in THF. The stirring solution was then exposed to UV light for 0.5 h before precipitating into diethyl ether. The polymer was isolated as a white powder and washed ×2 with diethyl ether (~ 1 g).

Solvent Casting of Standalone Polymer Electrolyte films: In a glovebox, to a solution of polymer (0.4 g) dissolved in anhydrous THF (4 ml), the required amount of LiTFSI was added. After 12 h stirring, the solution was poured into a Teflon mould and the solvent allowed to evaporate (24 h). The resulting standalone film was then dried under vacuum at 70 °C for at least 72 h or until TGA and NMR analysis indicated no residual solvent.

Synthesis of 2-Mercaptoethyl Phosphonic Acid (MEPA): 2-Mercaptoethyl phosphonic acid was synthesized according to the literature procedure.[3] Under a stream of nitrogen gas, triphenylmethanethiol (4.28 g, 15.4 mmol, 1 equiv.) was added to NaH (0.4 g, 15 mmol, 1 equiv.) in anhydrous THF (130 ml), yielding a yellow solution. (2-bromoethyl)phosphonic acid diethyl ester (2.5 ml, 15 mmol, 1 equiv.) was then added, and the solution was stirred for 1 h before adding water (~13 ml) to quench any excess NaH. The resulting mixture was concentrated on the rotary evaporated before adding water (50 ml) and extracting with 3 × 80 ml dichloromethane. The organic layer was concentrated and dried under vacuum. Trituration with 20 ml diethyl ether afforded a white powder, diethyl(2-tritylsulfanylethyl)phosphonate (5.0 g, 82 % yield). To remove the trityl protection group, the white powder was dissolved in trifluoroacetic acid (TFA), and triethylsilane was added dropwise to the rapidly stirring solution until the yellow colour disappeared and a white precipitate formed. The precipitate was removed by vacuum filtration, and the supernatant concentrated to yield a brown oil. The oil in a flask equipped with a Dean-Stark trap and condenser was hydrolyzed in refluxing 5 M HCl (75 ml) for 72 h. The aqueous layer was then washed with chloroform (2 × 100 ml) and concentrated *in vacuo* to afford MEPA as an off-white solid (1.1 g, 70 % yield).

Synthesis of $LMgCo(OAc)_2$ Catalyst. The macrocycle ligand, **HL2** was synthesized as per our previous literature reports.[4] The Mg(II)Co(II) heterodinuclear complex was synthesized as previously reported by our group.[5]

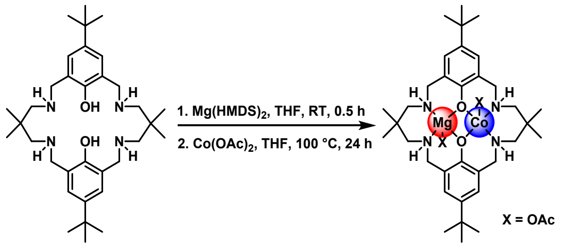

HL2 LMgCo(OAc)$_2$

Composite Cathode Fabrication and Cell Assembly. Polycrystalline NMC811 with 1 wt% $LiNbO_3$ coating, LPSCl (MSE Supplies), polymer electrolyte (10-50 mg) and carbon nanofiber (CNF, Merck) were mixed in a 70:23:5:2 wt% ratio by hand-grinding. The resulting powder was then cold-pressed together at 400 MPa with a layer of pure LPSCl and a composite of $Li_4Ti_5O_{12}$ (LTO), LPSCl, and CNF in a glovebox filled with Argon. The cells were assembled in a custom designed module with a PEEK mould and two stainless steel plungers. For control experiments without polymer a 70:27:2 wt% ratio was used.

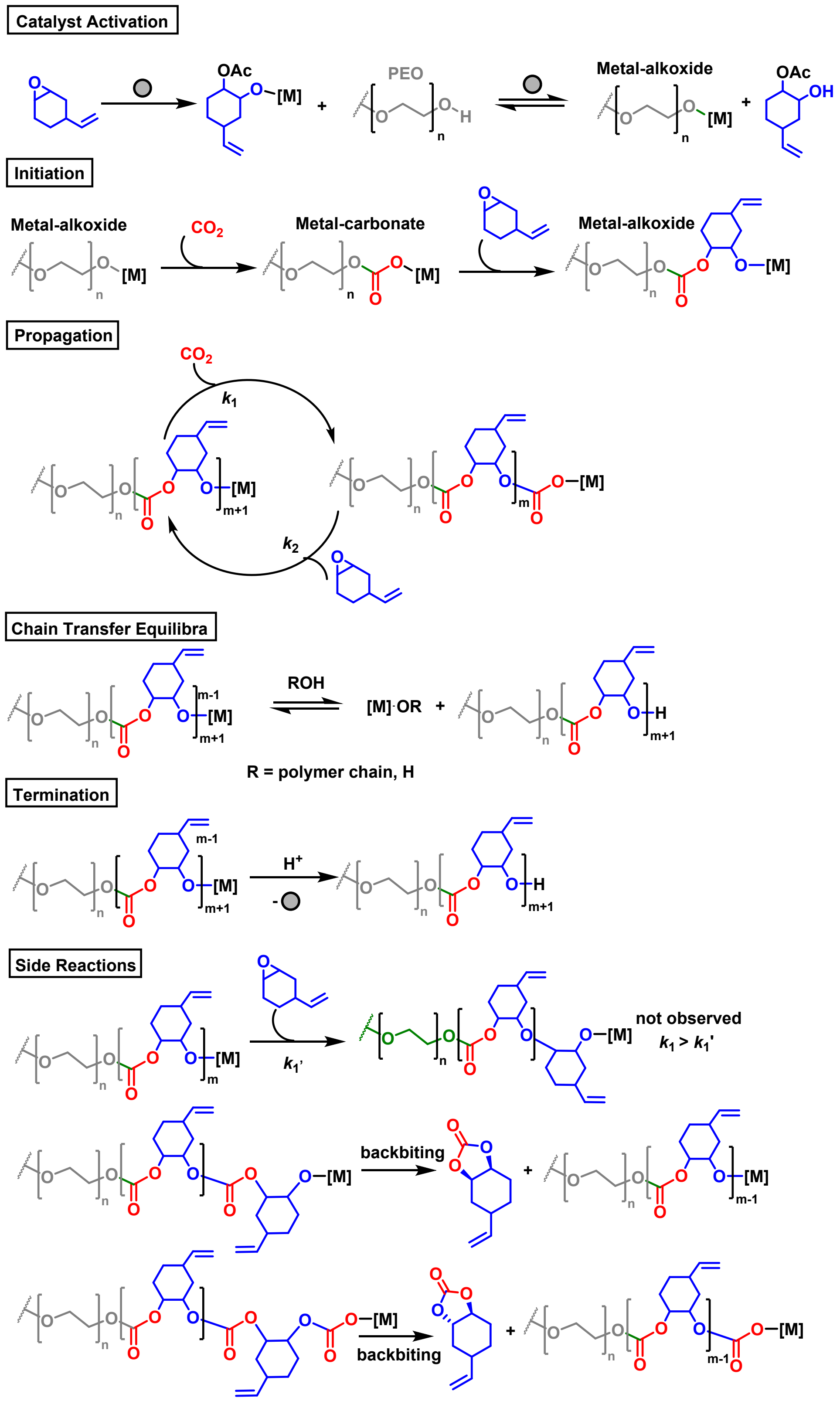

Fig. S1 Ring-Opening Copolymerization (ROCOP) mechanism. NB. Sequential ring-openings of epoxide monomer during propagation leading to polyether linkages (δ_H 3.45 ppm) as opposed to polycarbonate (δ_H 4.76 ppm) is not observed with this catalyst as CO_2 insertion into the metal alkoxide is faster than epoxide ring-opening. For simplicity, initiation and propagation are only illustrated from one end of the PEO macro-initiator.

Table S1. Details of PEO macroinitiators used in this work.

PEO (kg mol^{-1}) ^{a}	n ^{b}	$M_{n,SEC}$ (kg mol^{-1}) [$Đ$] ^{c}	T_m (°C) ^{d}	ΔH_f (J g^{-1}) ^{d}
1	23	1.2 [1.12]	35.6	88.04
3.4	76	4.2 [1.08]	60.6	186.11
8	182	11.0 [1.10]	61.7	194.04
35	795	28.7 [1.13]	65.1	156.04
100	2272	n.d.	68.3	179.93

^{a} As quoted by supplier. ^{b}number of EO repeat units based on polymer M_n and M_{EO}. $^{c}M_n$ recorded by SEC of samples in chloroform eluent, RI detector *vs* PS standards. $Đ$ = Dispersity (M_n/M_w). ^{d} Melting point and enthalpy recorded by DSC.

Table S2. Synthesis of PC-*b*-PEO-*b*-PC (CEC) triblock polymers.

CEC(n, f_{PC})^{a}	PC (wt%)^{b}	[Cat]:[PEO]:[vCHO]	[Cat] (mM)	Cat (mol%)^{c}	vCHO conv. (%)^{d}	T (h)	Triblock M_n (kg mol^{-1})^{e}	
							Theory	NMR
**CEC(795,0.07)**	7	1:20:600	0.44	0.2	45	0.5	37	37.6
**CEC(795,0.16)**	16	1:20:6000	0.44	0.02	11	2	41	41.7
**CEC(795,0.26)**	26	1:20:6000	0.44	0.02	34	6	52	47.0
**CEC(795,0.33)**	33	1:20:6000	0.44	0.02	41	7	56	51.5
**CEC(795,0.37)**	36	1:20:6000	0.44	0.02	50	8	60	55.0
**CEC(795,0.55)**	54	1:20:6000	0.44	0.02	96	12	83	76.5
**CEC(795,0.70)**	70	1:20:12000	0.22	0.01	82	24	119	115
**CEC(182,0.37)**	36	1:20:2400	3.2	0.04	30	3	14	12.5
**CEC(182,0.51)**	50	1:20:2400	3.2	0.04	50	5	18	16.0
**CEC(182,0.70)**	70	1:20:2400	3.2	0.04	74	7	23	27.0
**CEC(76,0.11)**	9	1:20:1000	8	0.1	6	0.2	4.0	4.0
**CEC(76,0.43)**	41	1:20:1000	8	0.1	23	0.5	5.3	6.0
**CEC(76,0.70)**	70	1:20:1000	8	0.1	62	1	8.5	11
**CEC(23,0.4)**	39	1:20:600	13.3	0.17	21	0.25	2.0	1.7
**CEC(23,0.78)**	77	1:20:600	13.3	0.17	78	1.0	4.6	4.4
**CEC(2272,0.27)**	27	1:20:12000	0.16	0.01	49	48	150	138

^{a} Nomenclature CEC(n, f_{PC}) where n = number of EO repeat units in PEO macroinitiator and f_{PC} = volume fraction of PC. f_{PC} = 1- f_{PEO} and $f_{PEO} = \frac{v_{PEO}}{v_{PEO} + \frac{M_{PC}}{M_C}\frac{M_{EO}}{M_{PEO}} v_{PC}}$ where M_{EO} and M_C are the molar masses of EO (44.05 g mol^{-1}) and vCHO/CO_2 (168.23 g mol^{-1}), respectively. M_{PEO} and M_{PC} are the number-averaged molecular weights of the PEO and PC blocks in kg mol^{-1}. Molar volumes v_{PEO}, v_{PC} were calculated by $v = M/\rho$ using densities (g cm^{-3}) of PC and PEO of 1.10 g cm^{-3} and 1.12 g cm^{-3}.[6] ^{b} Determined from purified polymer by relative ^{1}H NMR integration of PEO (3.64 ppm) vs PC (5.75 or 2.42 ppm) using PC repeat unit of 168.23 g mol^{-1}. ^{c} Catalyst loading (catalyst/epoxide). ^{d} vCHO conversion to PC determined from ^{1}H NMR analysis of crude reaction mixture. ^{e} Overall theoretical molar mass, M_n calculated based on initial monomer/initiator ratio, $M_{n,PEO}$ and conversion of vCHO to PC. M_n by NMR is determined by relative integration of the PEO and PC environments and taking into account $M_{n,PEO}$ of the macroinitiator (see Table S1).

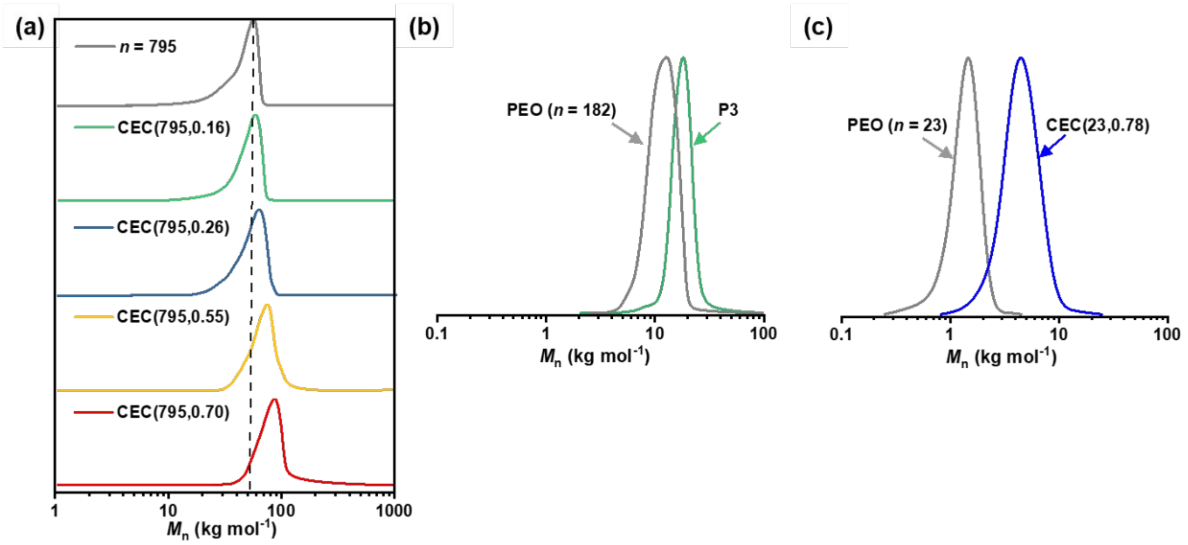

Fig. S2 Size Exclusion Chromatography (SEC). (a) Pure PEO macroinitiator (top) and below triblock polymers (CEC) with increasing polycarbonate outer block lengths. (b) Pure PEO macroinitiator (182 EO units) and CEC(182,0.37)/**P3**. (c) Pure PEO macroinitiator (23 EO units) and CEC(23,0.78). All SEC traces were measured in $CHCl_3$ eluent, RI detector vs narrow PS standards. NB. different x-axis scale for (a) cf (b) and (c).

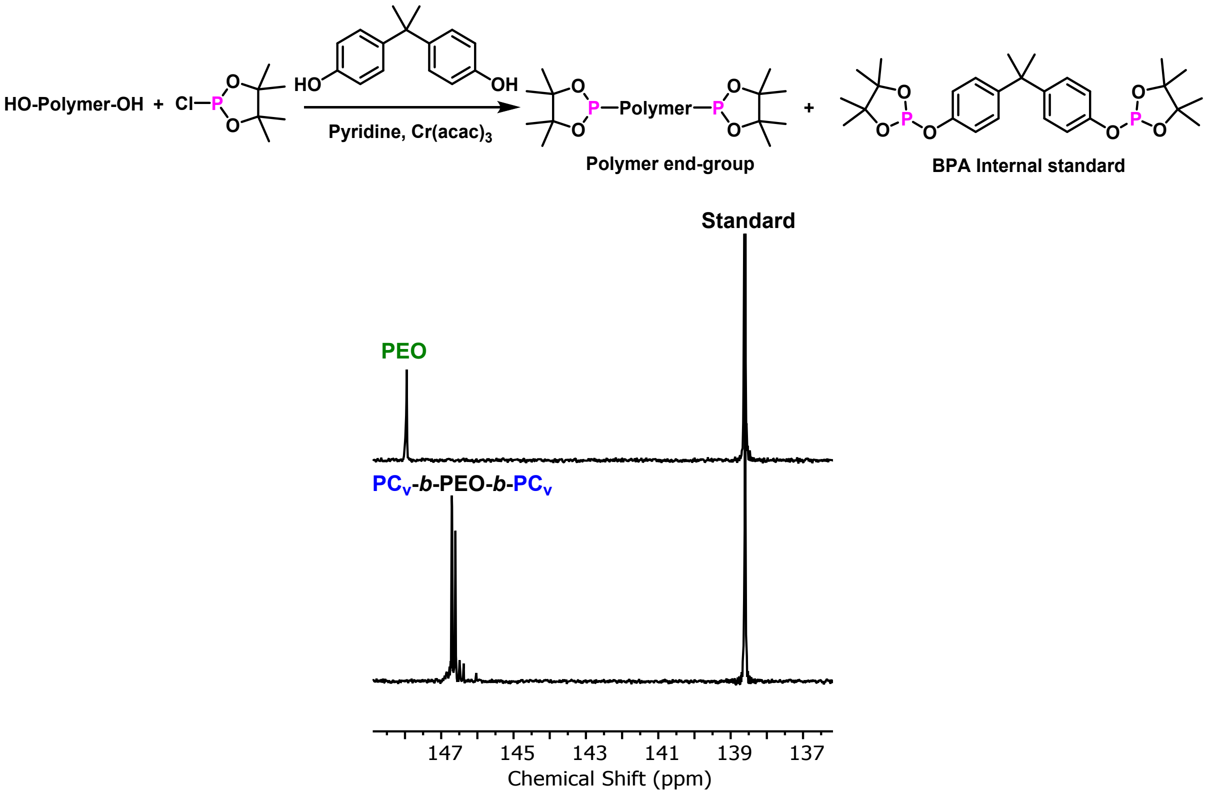

Fig. S3 Polymer end-group analysis by phosphorous test. Stacked $^{31}P\{^1H\}$ NMR spectra ($CDCl_3$) of PEO homopolymer (end-group 148 ppm) and CEC (795,0.70)/**P2** (146.4-146.7 ppm) after reaction of the –OH end groups with phosphorous reagent as shown. The latter is characteristic of PC end-groups, indicating no PEO end-groups remaining in the PC-PEO-PC triblock. The standard at 138.6 ppm is Bisphenol-A. End-group tests were conducted for all triblock polymers to check that only PC end-blocks were present.

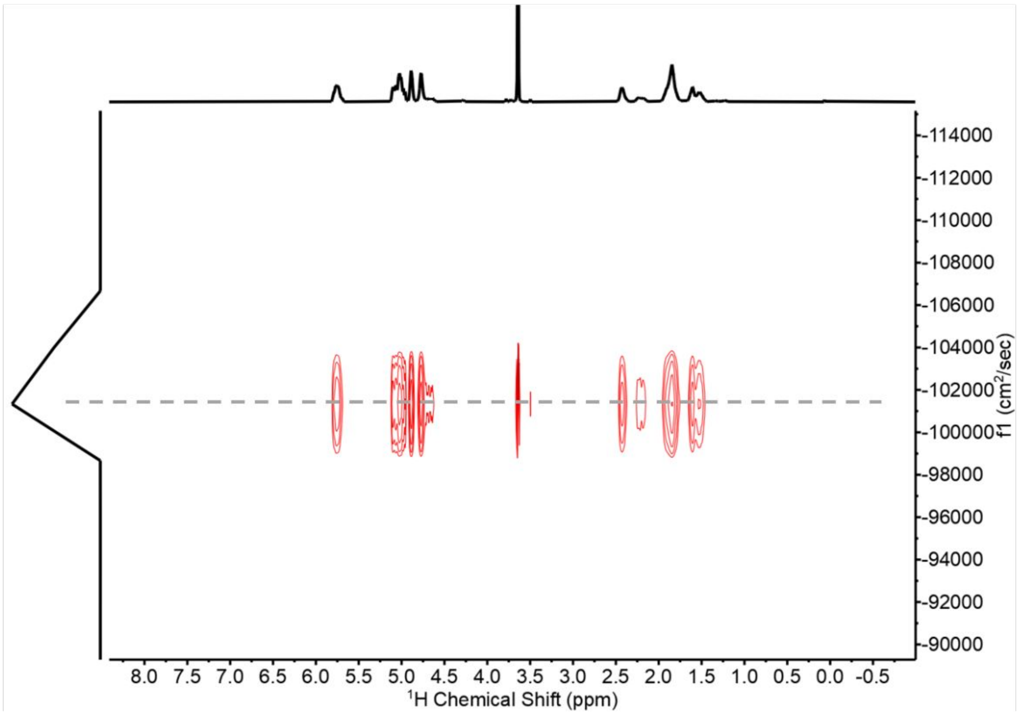

Fig. S4 DOSY NMR for CEC(182,0.51).

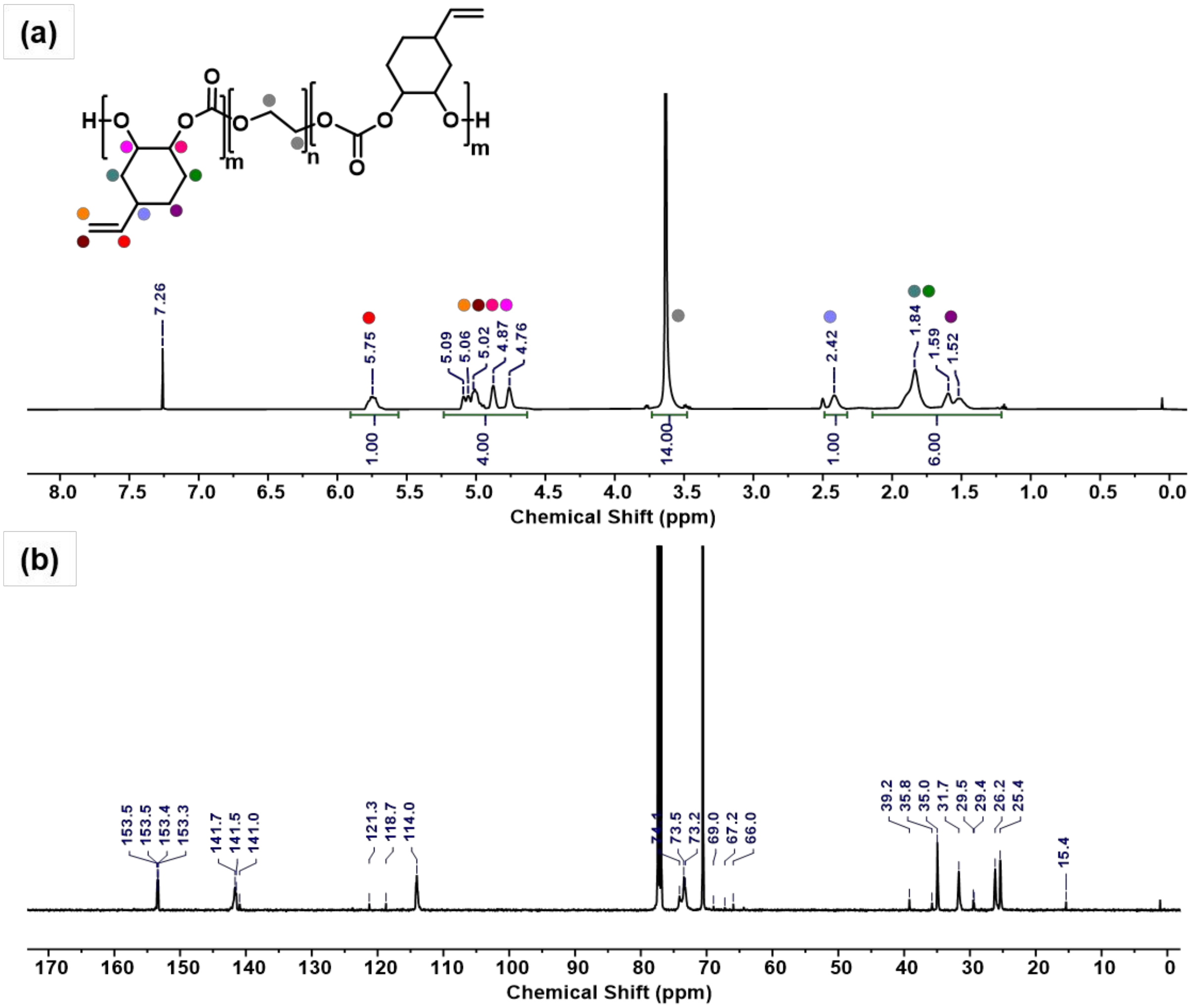

Fig. S5 Representative NMR spectra of purified PC-*b*-PEO-*b*-PC. (a) ^{1}H NMR Spectrum ($CDCl_3$) for CEC(795,0.55). The wt% PC was determined by relative integration of the vinyl proton (5.75 ppm) or methine (2.42 ppm) vs PEO (3.64 ppm). (b) $^{13}C\{^{1}H\}$ NMR ($CDCl_3$) for CEC(795,0.55).

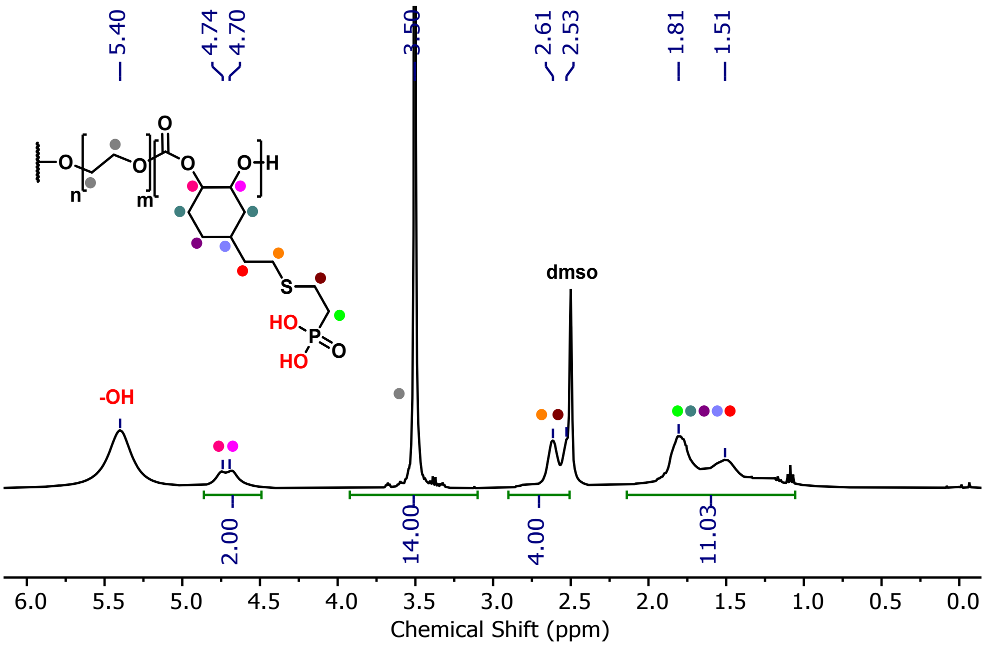

Fig. S6 NMR analysis of MEPA post-functionalized PC-PEO-PC triblock. (a) ^{1}H NMR (dmso-d_6) of CEC(795,0.55) with fully functionalized PC block.

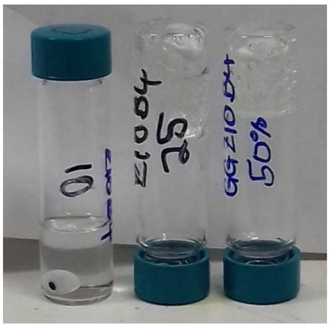

MEPA (%): 10, 25, 50 →

Fig. S7 Gel behaviour observed with higher MEPA content (wt%).

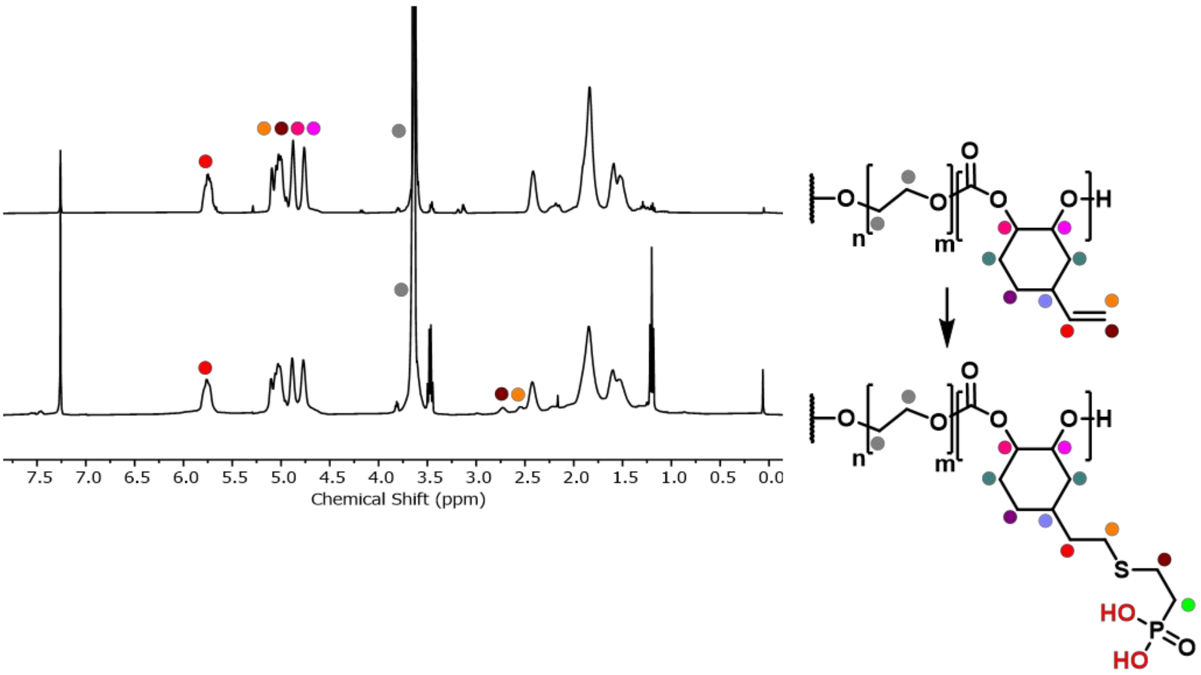

Fig. S8 ^{1}H NMR spectra ($CDCl_3$) confirming partial MEPA functionalization of PC. Top: unmodified CEC(795,0.55) and bottom: 6 wt% grafted MEPA (15% PC functionalization). Partial functionalization was confirmed by relative integration using the integrals for the vinyl protons (5.75 ppm), the PEO backbone (3.64 ppm) and new thio-ether signals (~2.72 ppm).

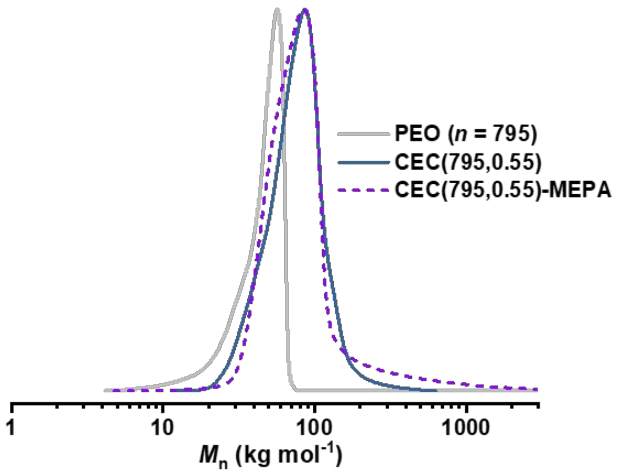

Fig. S9 SEC ($CHCl_3$ eluent) of CEC(795,0.55)-*g*-MEPA (3 wt%) confirming no cross-linking behaviour. The polymer molar mass remained similar, as expected for a partly functionalized backbone.

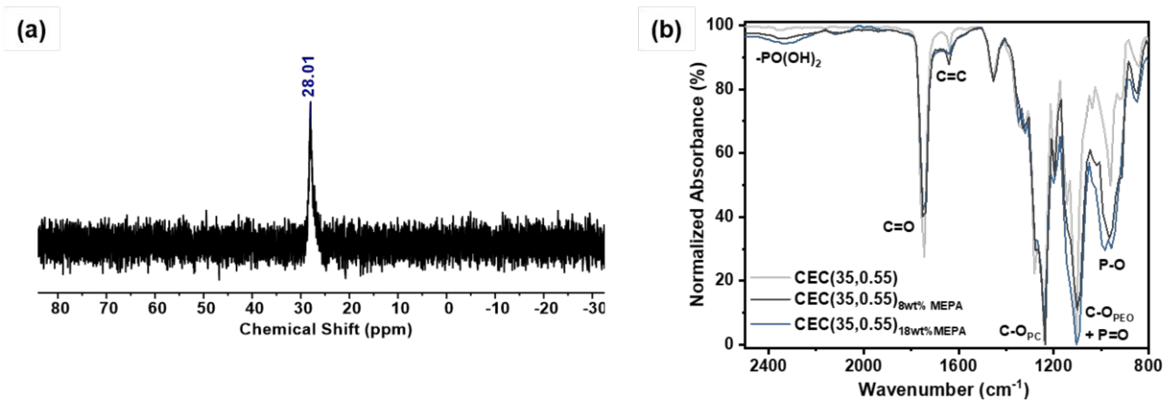

Fig. S10 Additional characterization of partially MEPA decorated polymers. (a) $^{31}P\{^{1}H\}$ NMR of purified partially post-functionalized triblock polymer. (b) FTIR for CEC(795,0.55).

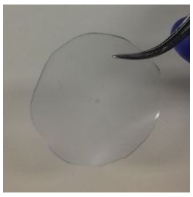

Fig. S11 Picture of standalone SPE film

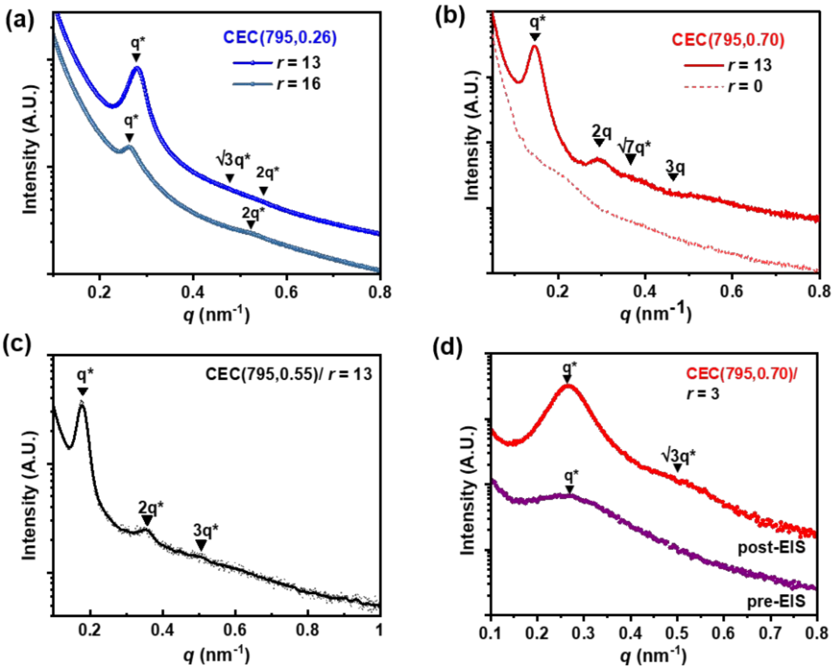

Fig. S12 Additional SAXS data. (a) CEC(795,0.26)/**P1** at different salt ratios. (b) CEC(795,0.70)/**P2** with and without LiTFSI. (c) CEC(795, 0.55) at *r* = 13. (d) CEC(795, 0.70)/*r* = 3 measured before and after EIS measurement (30 to 80 °C).

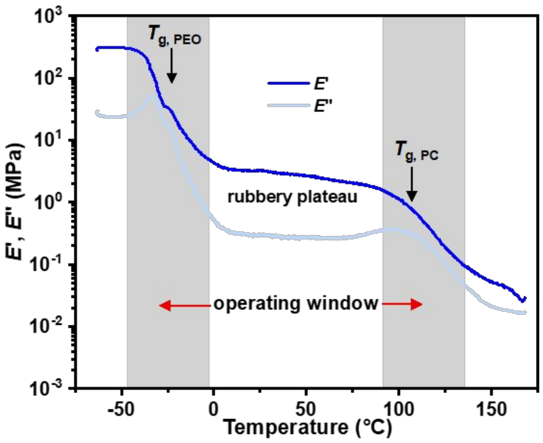

Fig. S13 Dynamic Mechanically Thermal Analysis (DMTA) conducted for P1/ *r* = 13. Experiments were conducted under tension at 0.1% strain, 1 Hz frequency from -60 to +180 °C at 3 °C min^{-1}.

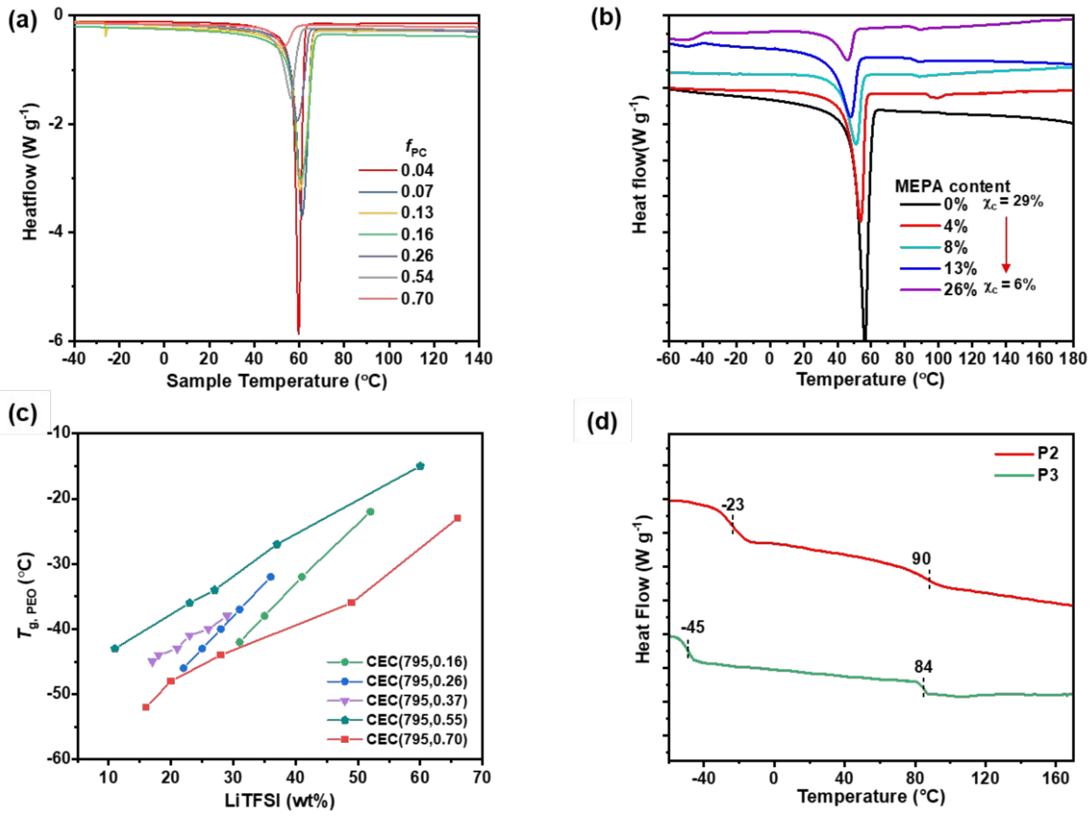

Fig. S14 Additional DSC data for polymers and polymer electrolytes. (a) Influence of f_{PC} on PEO crystallinity (χ_c) for $M_{n,PEO}$ = 35 kg mol^{-1} (n = 795) with no added lithium salt. The decrease in T_m (61 to 57 °C) and crystallinity (from 57 to 19 %) with increasing hard domain content is attributed to a confinement effect of the PEO mid-segment.[7] PEO crystallinity was calculated using ΔH_f = 214.6 J g^{-1} for 100% crystalline PEO. (b) Influence of grafted MEPA wt% on thermal properties for CEC(795,0.55). (c) $T_{g, PEO}$ as a function of lithium salt content for CEC triblock polymers with n = 795 and f_{PC} as labelled. (d) DSC traces for lead polymers, **P2** and **P3** at optimized salt ratios for ionic conductivity of r = 2 and 13, respecively (**P1**/r = 13 is shown in Figure 1c).

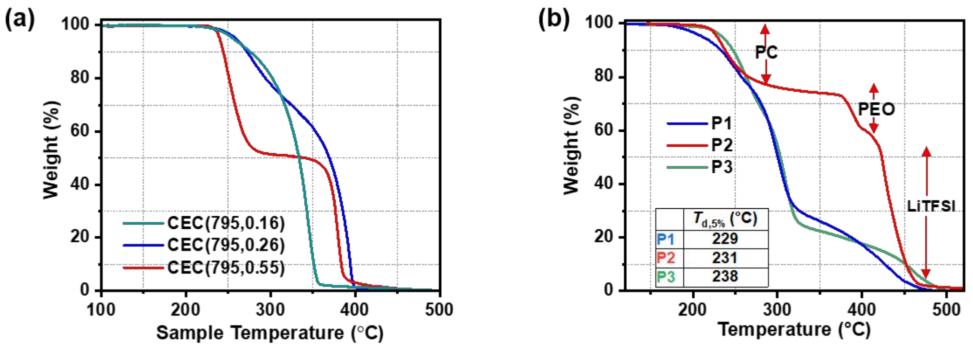

Fig. S15 TGA curves. (a) CEC(795, f_{PC}). (b) Polymer electrolytes **P1**, **P2** and **P3**.

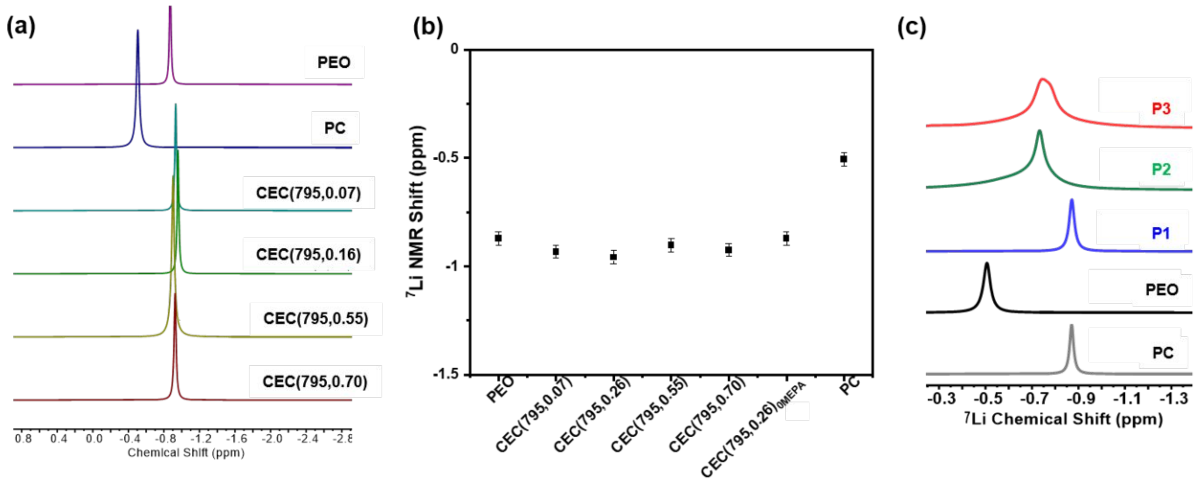

Fig. S16 ^{7}Li NMR($CDCl_3$) of polymer electrolytes. (a) Spectra of PEO (n= 795) and PC homopolymers (top) and CEC triblock polymers (bottom) with increasing PC block lengths. All samples contain salt ratio, r = 13. (b) Plot of ^{7}Li NMR shift for different polymers/r = 13 . (c) Spectra for **P1-P3**: **P1** = CEC(795,0.26)/ r = 13, **P2** = CEC(182,0.37)/ r = 13, **P3** = CEC(795, 0.79)/ r = 2.

Table S3. Surface energy measurements.

Polymer/ r = 13	wt% *g*-MEPA	Surface Free Energy (mJ m^{-2})
CEC(795,0.70)	6	40.5
CEC(182,0.37)	6	33.9
CEC(795,0.26)	0	48
CEC(795,0.26)	3	44
CEC(795,0.26)	6	39
NMC[8]	n.a	760 to 2270

Wetting of polymer on surface of cathode material: $\gamma_{polymer} < \gamma_{active\ material}$. Contact Angle Approach using Owens/Wendt Theory with water, glycol and diiodomethane.

$$\sigma_{SL} = \sigma_S + \sigma_L - 2(\sigma_L^D \sigma_S^D)^{1/2} - 2(\sigma_L^P \sigma_S^P)^{1/2}$$

$$\sigma_S = \sigma_{SL} + \sigma_L cos\theta$$

Where σ_L/σ_S = overall surface tension of the wetting liquid/overall surface energy of the solid, $\sigma_{L/S}{}^D$ = dispersive component of the surface tension of the wetting liquid/surface energy of the solid; $\sigma_{L/S}{}^P$ = polar component of the surface tension of the wetting liquid/surface energy of the solid; σ_{SL} = the interfacial tension between the solid and the liquid, and θ = the contact angle between the liquid and the solid.[9]

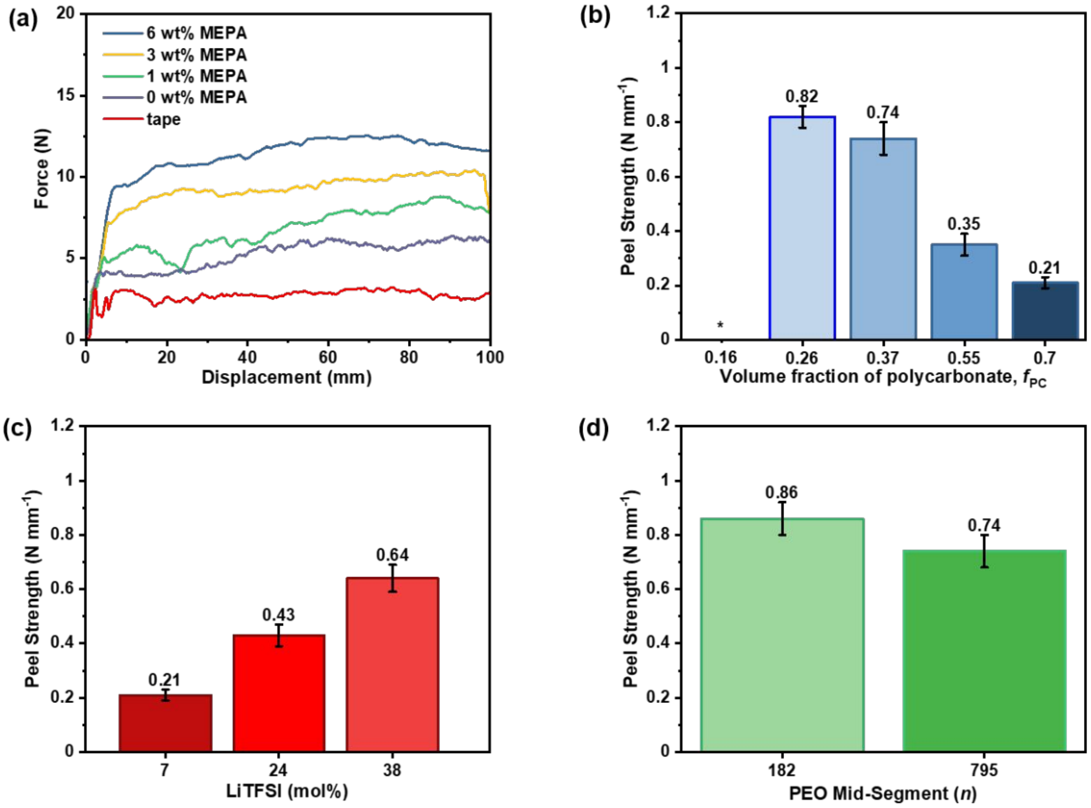

Fig. S17 Additional 180° peel tests on alumina. (a) Force vs displacement for triblocks CEC(795, 0.26) differing in grafted MEPA content (Figure 2b in article). Peel Strengths as a function of: (b) PC volume fraction at fixed n = 795 and r = 13. (c) LiTFSI ratio for CEC(795,0.70). 7, 24 and 38 mol% correspond to r = 13, 3 and 2, respectively. (d) PEO Segment length at r = 13 and f_{PC} = 0.37. * = tacky film unable to test.

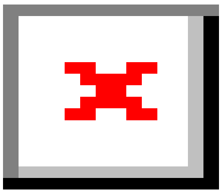

Fig. S18 SEM image of polymer-NMC composite. Polymer electrolyte (3 wt%) mixed with NMC811 cathode particles (10 μm).

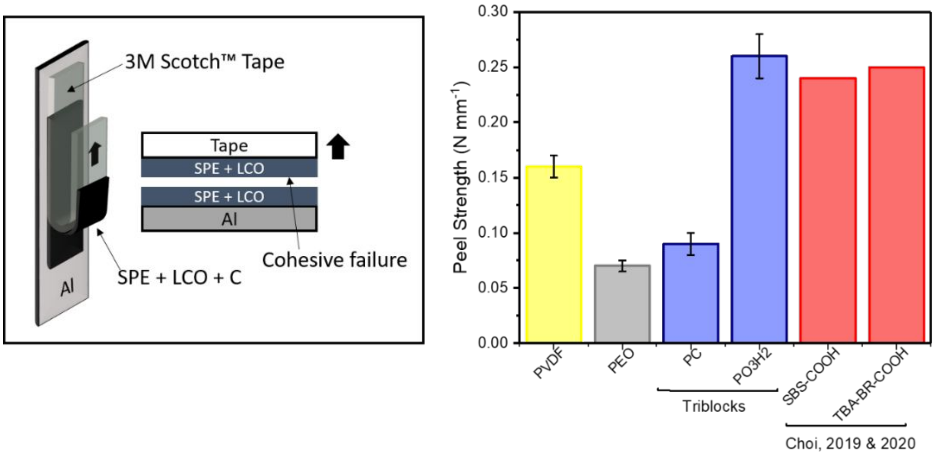

Fig. S19 180° Peel tests of composite cathode on Al current collector. Left: Schematic of the testing procedure following Choi *et al*.[10-11] Peel rate 305 mm min^{-1}. Right: Peel Strength measured adhesion of polymers as binders. A slurry of cathode material, SPE and C black (7:2:1 wt%) was prepared in anhydrous THF and applied via a doctor blade to a sheet of Aluminium foil current collector (wet film thickness = 80 µm). After drying in a vacuum oven at 60 °C for 72 hours, 180° Peel tests were performed. Under these conditions, the cohesive failure of the coating was examined not the adhesion to the aluminium current collector. The higher adhesion of PVDF compared to PEO is attributed to differences in molecular weight and thus adhesion by chain entanglement.

Table S4. SPE film thickness measurements

	**Film Thickness**
**CEC(795,0.37)/ salt ratio, *r* =**	**Digital Microscope (µm)**
18	79.9 ± 1.5
16	91.6 ± 1.2
13	92.2 ± 1.3
11	83.6 ± 1.2
9	130.8 ± 1.0

A Keyence VHX 7000 Digital Microscope (14 measurements over 2 cross-sections) was used to determine accurate film thicknesses for films < 150 µm.

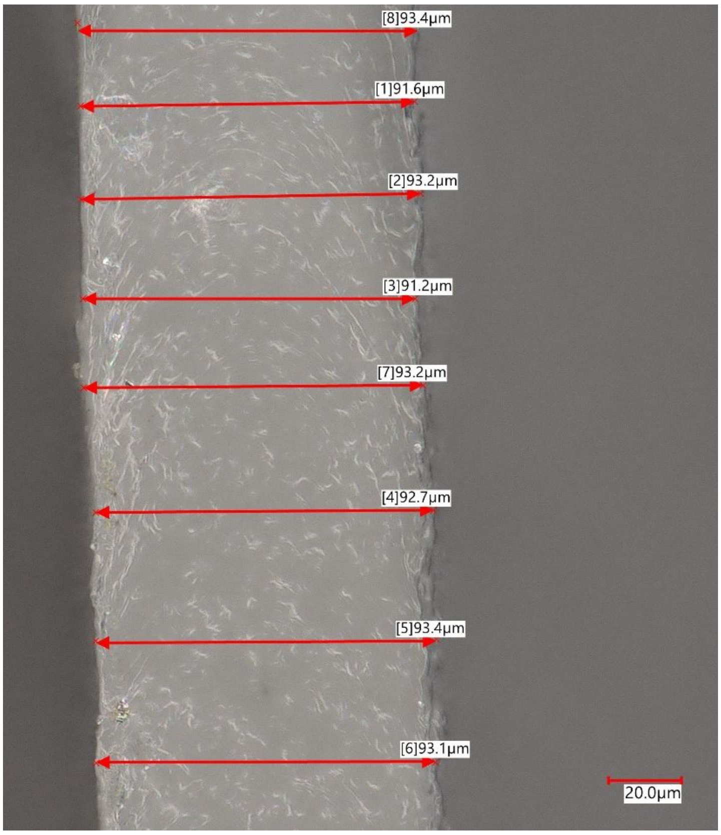

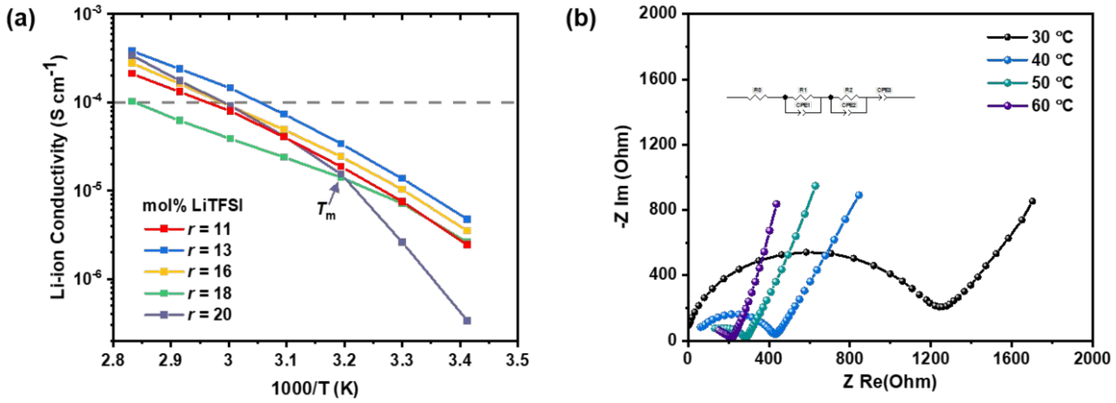

Fig. S20 Optimisation of LiTFSI content for CEC(795,0.37). (a) Li-ion conductivity as a function of temperature and salt ratio with gold blocking electrodes: Au|SPE|Au. The ‘bend’ in the data coincidences with a PEO melting transition observed by DSC. For PEO homopolymer, typically *r* = 10-12 is optimum. (b) Representative Nyquist curves (inset: equivlence circuit).

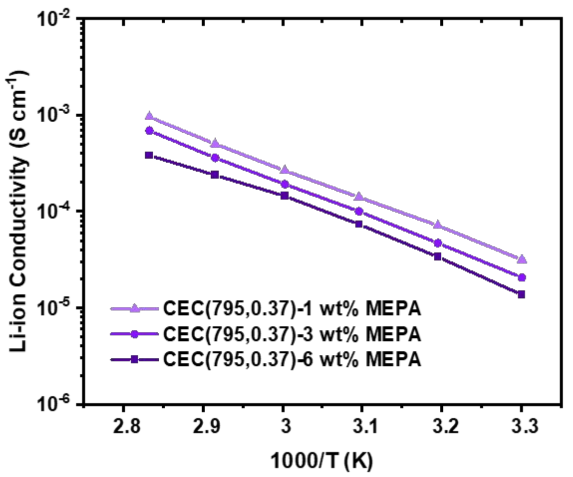

Fig. S21 Li-Ion conductivity as a function of MEPA wt%/ *r* = 13 .

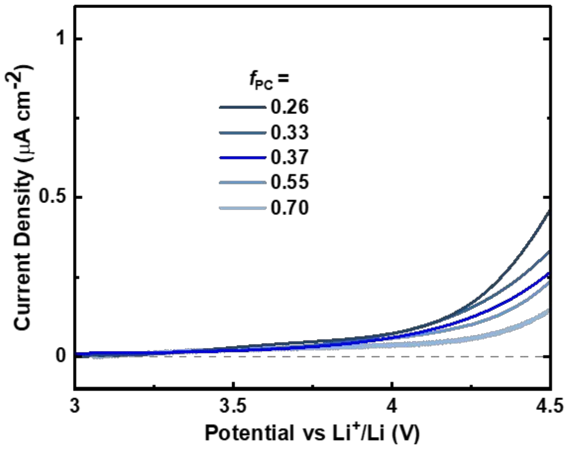

Fig. S22 Oxidative stability as a function of f_{PC}. *n* =795/ ***r* = 13**. Cell set-up: Li|SPE|SS (SS = stainless steel). Measurements conducted at RT from OCV to 6 V at 1 mV s^{-1} scan rate (see also Figure 5a).

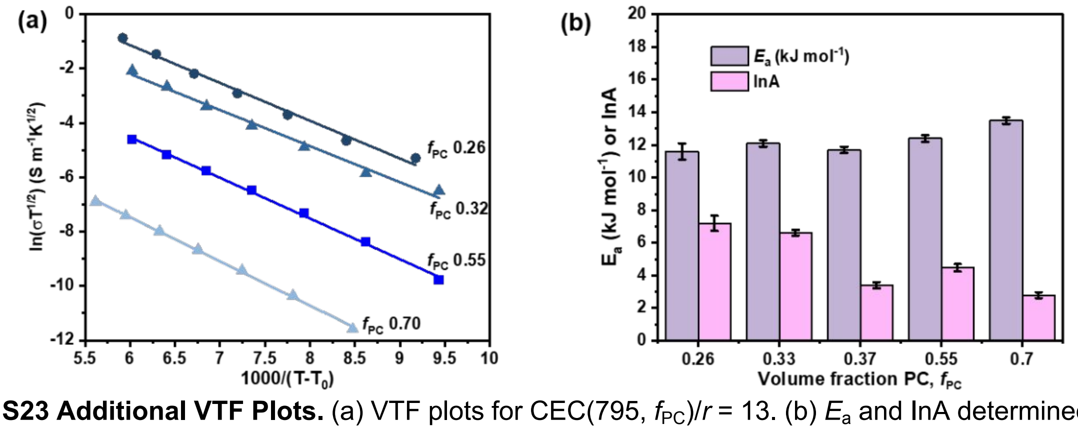

Fig. S23 Additional VTF Plots. (a) VTF plots for CEC(795, f_{PC})/*r* = 13. (b) E_a and lnA determined from VTF plots.

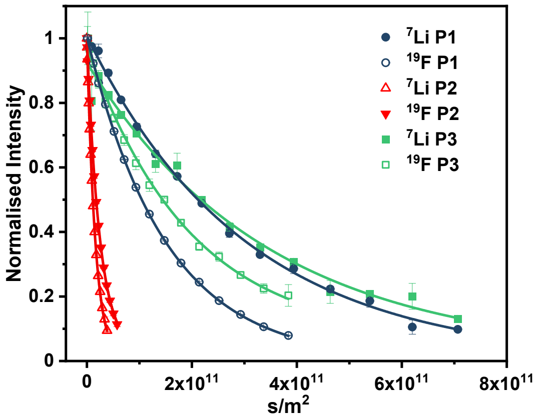

Fig. S24 Diffusion PFG Decays.

Table S5. Diffusion PFG-NMR

Polymer	^{7}Li Diff ($\times 10^{-12}$ m^2 s^{-1})	Error $\times 10^{-12}$	^{19}F Diff ($\times 10^{-12}$ m^2 s^{-1})	Error $\times 10^{-12}$
**P1**	3.344	0.1071	6.655	0.03909
**P2**	62.48	0.5418	38.81	0.5397
**P3**	2.824	0.3006	4.535	0.2369

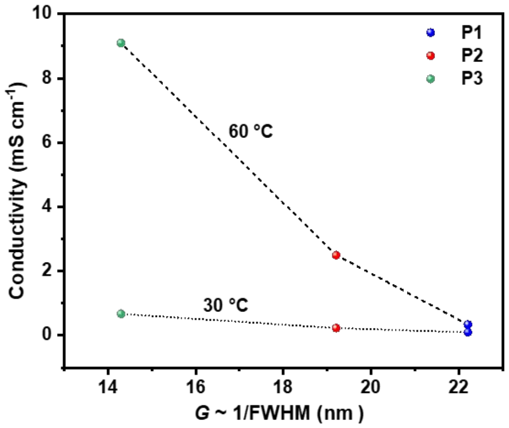

Fig. S25 Plot of 1/FWHM from SAXS (*q) vs ionic conductivity for P1, P2, P3.**

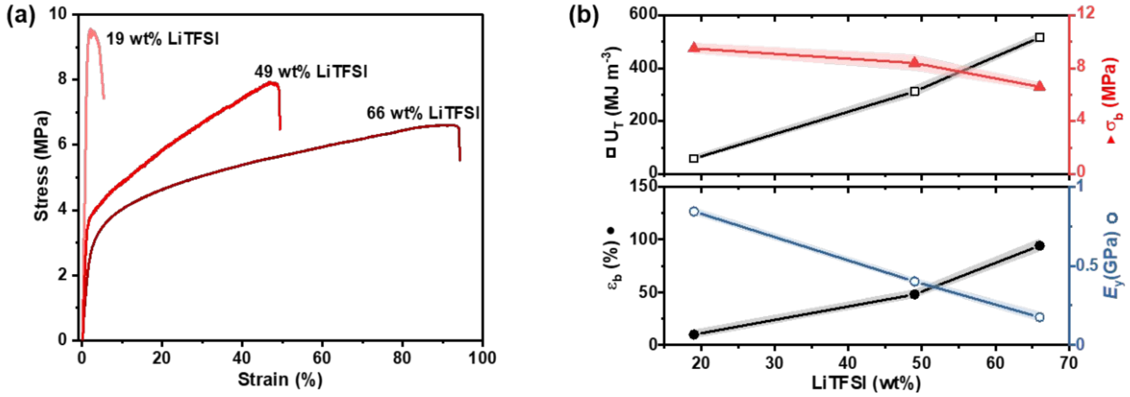

Fig. S26. Toughening of P2 with salt ratio. (a) Representative tensile stress-strain curves (10 mm min^{-1} strain rate. 19, 49 and 66 wt% LiTFSI correspond to r = 13, 3 and 2, respectively (7, 24 and 34 mol%). (b) Plots of tensile toughness (area under stress-strain curve), elongation at break, tensile strength and Young's Modulus as a function of salt content.

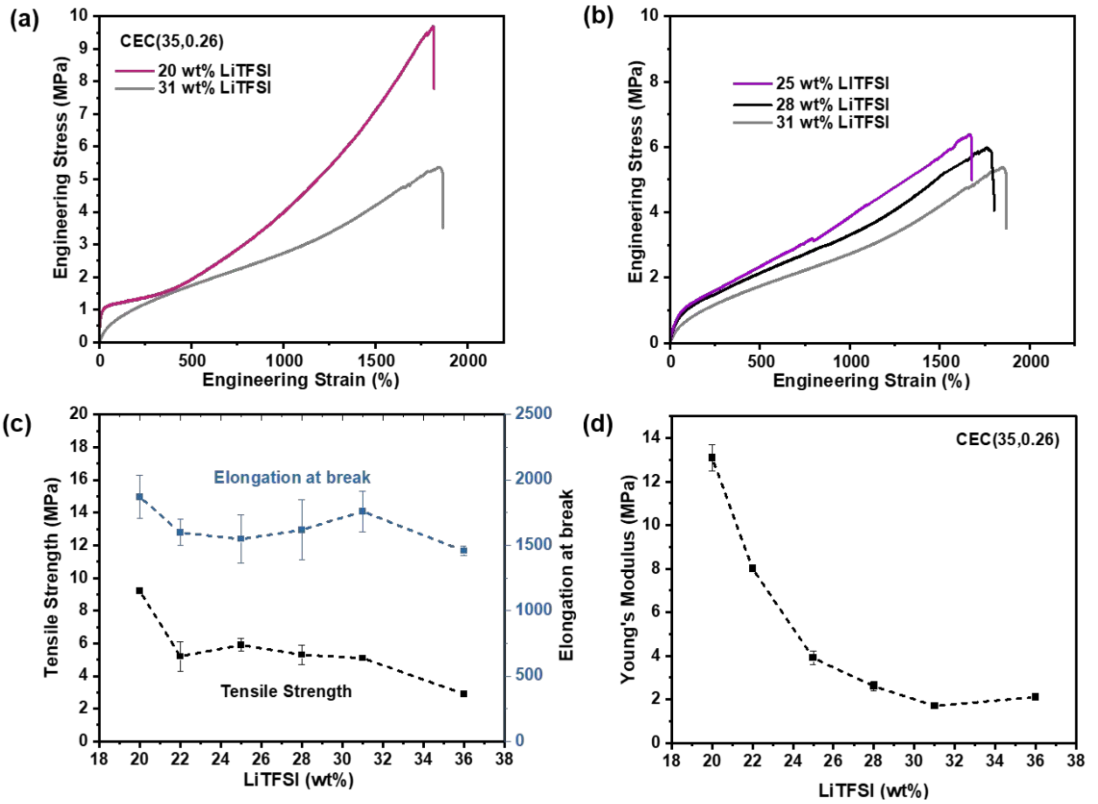

Fig. S27 Additional stress-strain curves for P1. (a)-(b) Varying salt ratios. 20 wt% LiTFSI (r = 18) contains semi-crystalline PEO whereas all others are amorphous by DSC. (c) Tensile strength and elongation at break as a function of LiTFSI, r = 13 corrosponds to 28 wt % LiTFSI. (d) Young's Modulus as a function of LiTFSI.

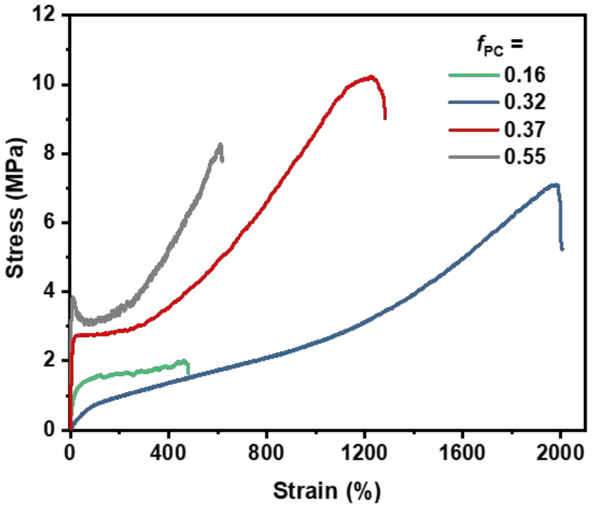

Fig. S28 Mechanical properties as a function of PC content. Representative tensile stress-stain curves for CEC(795, f_{PC}), r = 13.

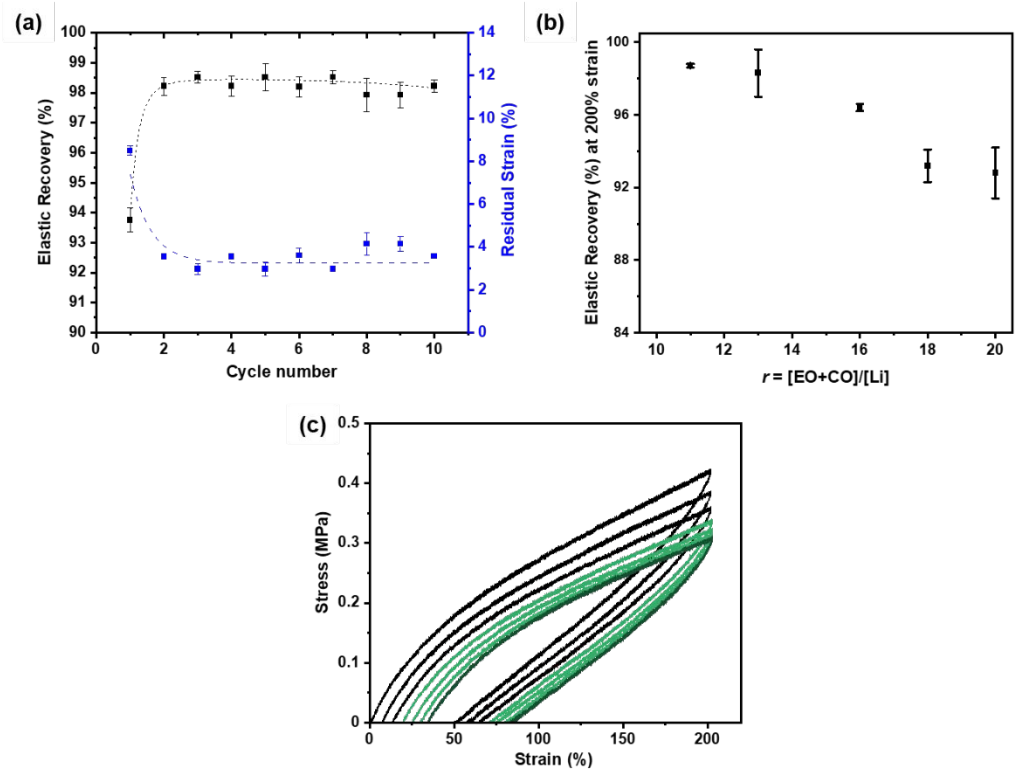

Fig. S29 Additional elastic recovery data. (a) Elastic recovery and residual strain as a function of cycle number for **P1**. (b) Elastic recovery for **P1** as a function of lithium salt content. (c) Elastic recovery for **P3**: 80.7 ± 0.4 %. All Measurements were conducted to 200 % strain under tension at a strain rate of 10 mm min^{-1}.

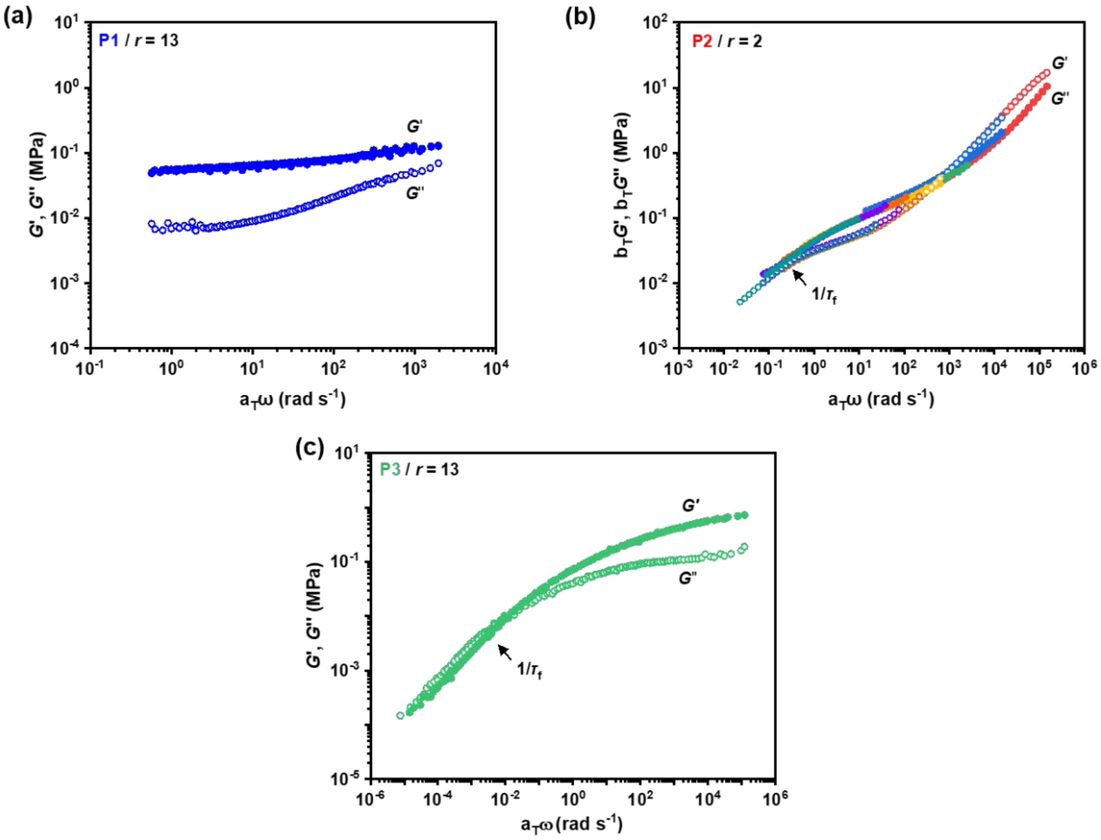

Fig. S30 Time-Temperature Superposition (Williams–Landel–Ferry, WLF theory) master curves for Storage and loss moduli (*G*', *G*'') at a reference temperature (T_{ref}) of 60 °C. WLF equation = $In(a(T)) = \frac{-C1\,(T-Tref)}{C2\,+(T-Tref)}$. (a) **P1**. WLF parameters (R^2 = 0.99): c1 = 0.27, c2 = 61.8 K. (b) **P2**. WLF parameters (R^2 > 0.99): c1 = 5.76, c2 = 102.2 K. (c) **P3**. WLF parameters (R^2 = 0.99): c1 = 4.24, c2 = 88.2 K. All measurements were in the linear viscoelastic region (as determined by amplitude sweeps). Master curves were generated from frequency sweeps at T = 30 to 80 °C, 10 °C intervals.

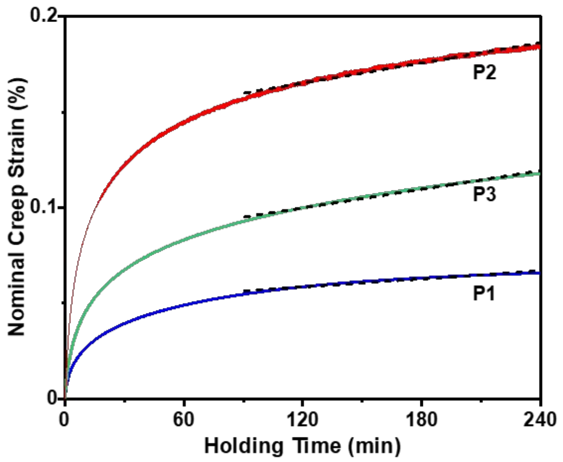

Fig. S31 Creep experiments. Compressive strain as a function of time conducted on polymer electrolyte discs at 60 °C under an applied 1 MPa load. For comparison, the strain for each of **P1- P3** has been normalized to the instantaneous elastic strain at 1 MPa load. Creep rates were determined from the steady state creep (gradient of dotted lines).

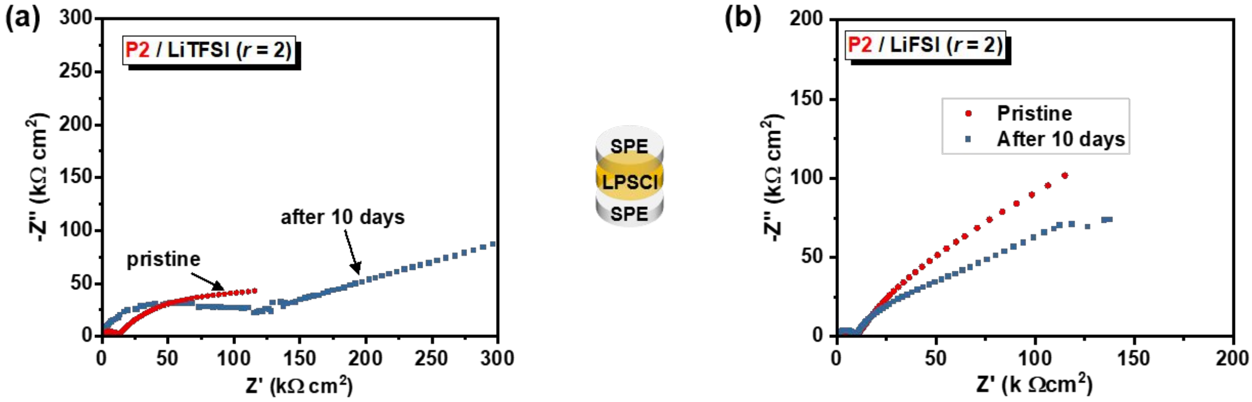

Fig. S32 Interfacial resistance for P2 with (a) LiTFSI and (b) LiFSI.

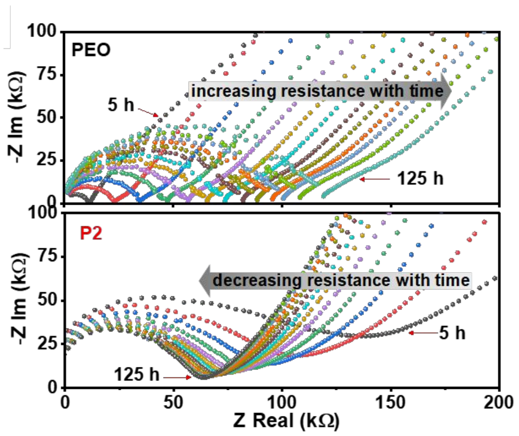

Fig. S33 P2/LPSCl Interfacial resistance. Nyquist curves measured at RT for **P2**|LPSCl|**P2** held at 60 °C for defined time periods (Figure 5b in article).

Table S6. Ionic conductivity and mechanical parameters of P1-P3 with LiFSI.[a]

Polymer Electrolyte	σ ($\times 10^{-3}$ S cm^{-1})[b]		E_y (MPa)
	30 °C	60 °C	
**P1**	0.13	0.35	2.6 ± 0.1
**P2**	0.25	2.9	183 ± 2.3
**P3**	0.74	10.4	1.7± 0.1

[a] The same salt loadings of LiFSI were used as for LiTFSI i.e **P1**/*r* = 13, **P2**/*r* = 2 and **P3**/ *r* = 13. Polymer electrolyte films were prepared exactly as for LiTFSI/polymer films by solvent casting from THF and drying at RT followed by in a vacuum oven at 70 °C until TGA and NMR indicated no residual solvent. [b]Ionic conductivity was measured by EIS as described previously: Au|SPE|Au. [c] Young's Modulus from uniaxial tensile testing (10 mm min^{-1} strain rate), RT measurements. Error bars represent standard deviation, *N* = 3.

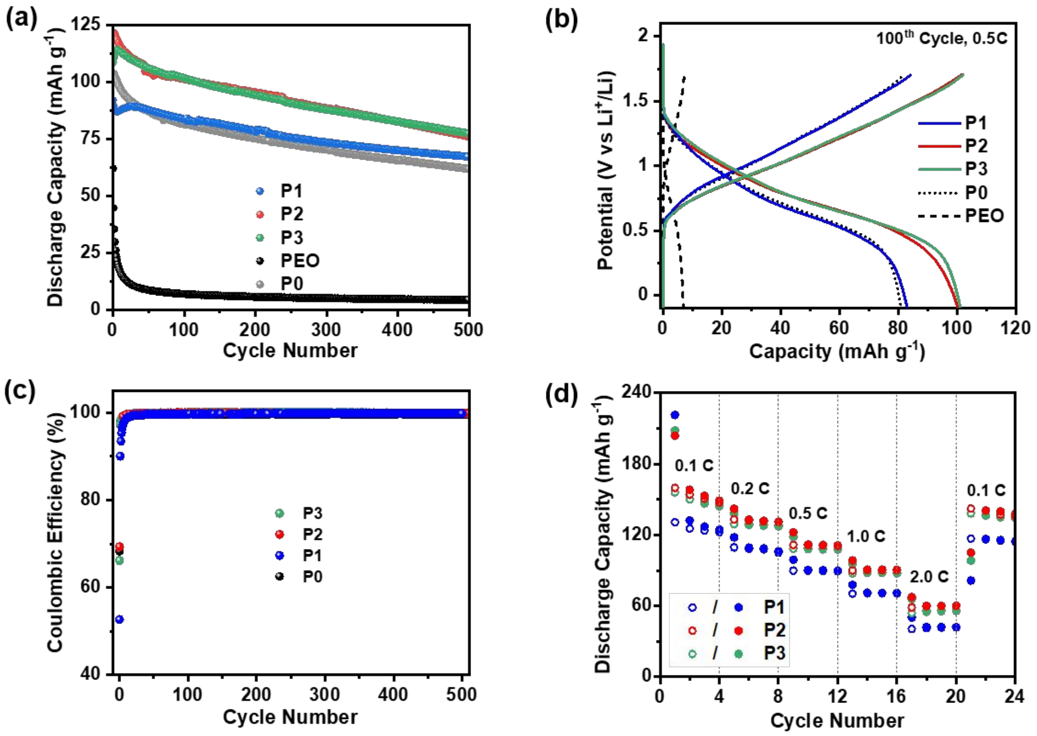

Fig. S34 Additional cell performance data. (a) Discharge capacity vs cycle number for LTO|LPSCl|NMC-SPE composite at 60 °C, 0.5C (Figure 5c). (b) Charge-discharge voltage profiles at 100th cycle. (c) Coulombic Efficiency vs cycle number. (d) Rate capability. Cell cycled at different C-rates.

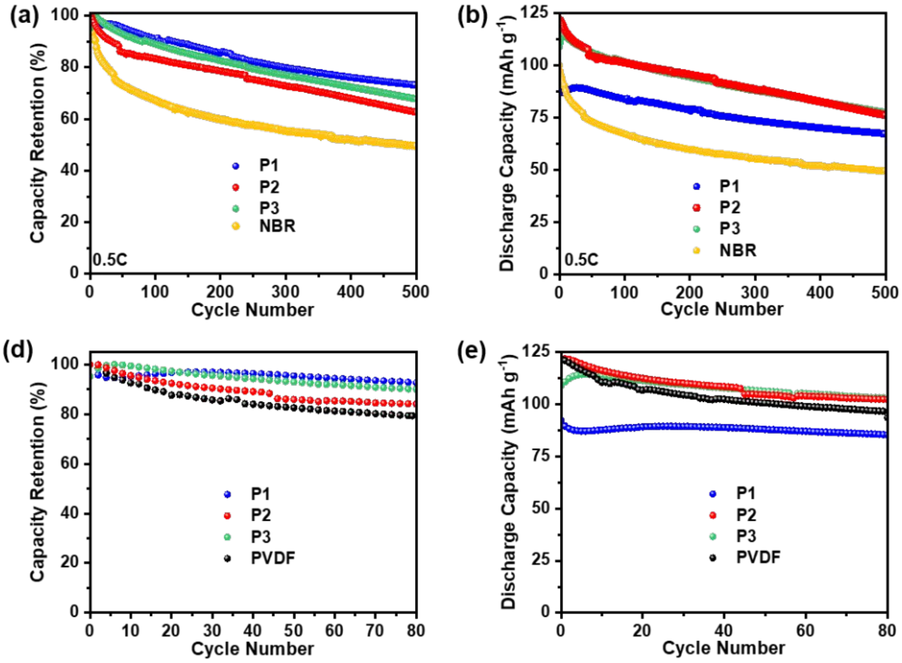

Fig. S35 Cell performance compared to traditional binders. (a)-(b) Capacity retention and discharge capacity vs cycle number using NBR binder in composite cathode compared to **P1-P3** polymer electrolytes. (c)-(d) Use of PVDF binder. All cells were cycled at 60 °C, 0.5C-rate. Composite cathodes with NBR or PDVF were prepared exactly as for **P1-P3** (described above and in text).

References

(1) Spyros, A.; Argyropoulos, D. S.; Marchessault, R. H. A Study of Poly(hydroxyalkanoate)s by Quantitative 31P NMR Spectroscopy: Molecular Weight and Chain Cleavage. *Macromolecules* **1997**, *30* (2), 327-329.

(2) Asakura, R.; Duchêne, L.; Kühnel, R.-S.; Remhof, A.; Hagemann, H.; Battaglia, C. Electrochemical Oxidative Stability of Hydroborate-Based Solid-State Electrolytes. *ACS Appl. Energy Mater.* **2019**, *2* (9), 6924-6930.

(3) Aldakov, D.; Sajjad, M. T.; Ivanova, V.; Bansal, A. K.; Park, J.; Reiss, P.; Samuel, I. D. W. Mercaptophosphonic acids as efficient linkers in quantum dot sensitized solar cells. *J. Mater. Chem. A* **2015**, *3* (37), 19050-19060.

(4) Sulley, G. S.; Gregory, G. L.; Chen, T. T. D.; Peña Carrodeguas, L.; Trott, G.; Santmarti, A.; Lee, K.-Y.; Terrill, N. J.; Williams, C. K. Switchable Catalysis Improves the Properties of CO2-Derived Polymers: Poly(cyclohexene carbonate-b-ε-decalactone-b-cyclohexene carbonate) Adhesives, Elastomers, and Toughened Plastics. *J. Am. Chem. Soc.* **2020**, *142* (9), 4367-4378.

(5) Deacy, A. C.; Kilpatrick, A. F. R.; Regoutz, A.; Williams, C. K. Understanding metal synergy in heterodinuclear catalysts for the copolymerization of CO(2) and epoxides. *Nat. Chem.* **2020**, *12* (4), 372-380.

(6) Loo, W. S.; Balsara, N. P. Organizing thermodynamic data obtained from multicomponent polymer electrolytes: Salt-containing polymer blends and block copolymers. *J. Polym. Sci. Part B: Polym. Phys.* **2019**, *57* (18), 1177-1187.

(7) Beaudoin, E.; Phan, T. N. T.; Robinet, M.; Denoyel, R.; Davidson, P.; Bertin, D.; Bouchet, R. Effect of Interfaces on the Melting of PEO Confined in Triblock PS-b-PEO-b-PS Copolymers. *Langmuir* **2013**, *29* (34), 10874-10880.

(8) Qian, G.; Zhang, Y.; Li, L.; Zhang, R.; Xu, J.; Cheng, Z.; Xie, S.; Wang, H.; Rao, Q.; He, Y.; et al. Single-crystal nickel-rich layered-oxide battery cathode materials: synthesis, electrochemistry, and intra-granular fracture. *Energy Storage Mater.* **2020**, *27*, 140-149.

(9) Stein, G. E.; Laws, T. S.; Verduzco, R. Tailoring the Attraction of Polymers toward Surfaces. *Macromolecules* **2019**, *52* (13), 4787-4802.

(10) Lee, K.; Lee, J.; Choi, S.; Char, K.; Choi, J. W. Thiol–Ene Click Reaction for Fine Polarity Tuning of Polymeric Binders in Solution-Processed All-Solid-State Batteries. *ACS Energy Lett.* **2019**, *4* (1), 94-101.

(11) Lee, J.; Lee, K.; Lee, T.; Kim, H.; Kim, K.; Cho, W.; Coskun, A.; Char, K.; Choi, J. W. In Situ Deprotection of Polymeric Binders for Solution-Processible Sulfide-Based All-Solid-State Batteries. *Adv. Mater.* **2020**, *32* (37), 2001702.
